# Through the eye of a Gobi khulan – Application of camera collars for ecological research of far-ranging species in remote and highly variable ecosystems

**DOI:** 10.1371/journal.pone.0217772

**Published:** 2019-06-04

**Authors:** Petra Kaczensky, Sanchir Khaliun, John Payne, Bazartseren Boldgiv, Bayarbaatar Buuveibaatar, Chris Walzer

**Affiliations:** 1 Norwegian Institute of Nature Research, Trondheim, Norway; 2 Research Institute of Wildlife Ecology, University of Veterinary Medicine Vienna, Vienna, Austria; 3 Ecology Group, Department of Biology, National University of Mongolia, Ulaanbaatar, Mongolia; 4 Wildlife Conservation Society, Mongolia Program, Ulaanbaatar, Mongolia; Universita degli Studi di Trento, ITALY

## Abstract

The Mongolian Gobi-Eastern Steppe Ecosystem is one of the largest remaining natural drylands and home to a unique assemblage of migratory ungulates. Connectivity and integrity of this ecosystem are at risk if increasing human activities are not carefully planned and regulated. The Gobi part supports the largest remaining population of the Asiatic wild ass (*Equus hemionus*; locally called “khulan”). Individual khulan roam over areas of thousands of square kilometers and the scale of their movements is among the largest described for terrestrial mammals, making them particularly difficult to monitor. Although GPS satellite telemetry makes it possible to track animals in near-real time and remote sensing provides environmental data at the landscape scale, remotely collected data also harbors the risk of missing important abiotic or biotic environmental variables or life history events. We tested the potential of animal born camera systems (“camera collars”) to improve our understanding of the drivers and limitations of khulan movements. Deployment of a camera collar on an adult khulan mare resulted in 7,881 images over a one-year period. Over half of the images showed other khulan and 1,630 images showed enough of the collared khulan to classify the behaviour of the animals seen into several main categories. These khulan images provided us with: i) new insights into important life history events and grouping dynamics, ii) allowed us to calculate time budgets for many more animals than the collared khulan alone, and iii) provided us with a training dataset for calibrating data from accelerometer and tilt sensors in the collar. The images also allowed to document khulan behaviour near infrastructure and to obtain a day-time encounter rate between a specific khulan with semi-nomadic herders and their livestock. Lastly, the images allowed us to ground truth the availability of water by: i) confirming waterpoints predicted from other analyses, ii) detecting new waterpoints, and iii) compare precipitation records for rain and snow from landscape scale climate products with those documented by the camera collar. We discuss the added value of deploying camera collars on a subset of animals in remote, highly variable ecosystems for research and conservation.

## Introduction

The Mongolian Gobi-Eastern Steppe Ecosystem is one of the largest remaining natural drylands and home to a unique assemblage of migratory ungulates, a rapidly disappearing biological phenomenon [1, 2]. Connectivity and integrity of this ecosystem are at risk if increasing human activities are not carefully planned and regulated [3–6]. The Gobi part supports the largest remaining population of the Asiatic wild ass (*Equus hemionus*; locally called “khulan”) globally assessed as “Near Threatened” by the IUCN Red List [7, 8]. Individual khulan roam over areas of thousands of square kilometers and the scale of their movements is among the largest described for terrestrial mammals, making them particularly vulnerable to an increase in the human footprint [9, 10] and notoriously difficult to monitor. The drivers of these large scale, nomadic movements are not fully understood, but variability in pasture productivity, the need to regularly access water, catastrophic events, and human activity have all been shown to influence khulan movement in the Gobi [9, 11–13].

GPS satellite telemetry now makes it possible to track animals in near-real time and largely uncompromised by environmental factors or observer bias. In combination with remote sensing weather and habitat data, it has allowed us to greatly expand our understanding of space use and movement patterns of wildlife globally [14]. Acceleration sensors integrated in GPS satellite collars have also become standard and allow inferences about the body posture or behavioural state of the animal [15]. However, remotely collecting data also harbors the risk of missing important abiotic or biotic environmental variables or life history events which may constitute important covariates for understanding time, cohort, or state-specific resource needs [16, 17]. Furthermore, correctly assigning activity sensor data to behavioural states requires ground-truthing of sensor values with behavioural observations [18–20].

The use of remote cameras has become increasingly popular, not only to detect and count species, but also to record their behaviour [21]. While camera traps may capture the behaviour of many individuals at a certain location, cameras attached to individual animals (animal-born camera systems, subsequently referred to as “camera collars”) can provide regular and direct insights into an individual’s interaction with peers, other species, and the environment [22–24]. By combining GPS satellite technology, collar sensor data, and image information from stills or videos, some of the limitations of remote monitoring can be overcome (also see [25]). The potential of camera collars to add to our understanding of far-ranging, elusive mammals and their environment seems enormous and researchers have just started to used camera collars to detect novel behaviours, diet choice and prey selection, habitat type preferences, and to calibrate acceleration sensors [22, 23, 26–28].

We tested the application of camera collars for khulan in the Mongolian Gobi, focusing on a set of six potential applications where we expected to gain additional insights from still images:

1) Currently our assessment of the body condition or reproductive state of a GPS tagged khulan is temporally restricted to the capture event. We have no way of assessing if the animals we are tracking are compromised by injury or disease, how long a female remains associated with a foal present during capture, or whether she has a new foal in the following year. We expected images to allow us to detect birth of foals as well as death or separation events from foals in female khulan.

2) Khulan social organization is thought to follow a fission-fusion dynamic where the only stable association is between a female and her foal [29]. Khulan tend to occur in small groups of 2–5 animals, but at times form aggregations of several hundred animals [7, 8, 30]. Remotely following small numbers of GPS-tagged khulan provides a limited opportunity to assess aggregation and grouping dynamics. Currently all locations of collared khulan are assumed to equally represent the movements of the overall khulan population. In reality, a collared animal may be alone, in small or large aggregations with other khulan. We expected images to allow us to assign a grouping index to daily locations and identify areas which attract large numbers of animals to be used as covariates in habitat use modelling.

3) We are still only at the beginning of understanding what is driving movements and population dynamics in khulan. So far, analysis has been primarily directed towards finding large-scale temporal and spatial patterns. However, subtler patterns may be lost if different life-stage events or behavioural states require different habitats (e.g. resting and feedings as compared to just travelling). It is very difficult to assess the strength of this kind of correlation without detailed behavioural data. Given the large range of khulan, the remoteness of the area and the large flight distances we see a huge potential of camera collar images to supplement opportunistic observations. We further expected camera collar images to allow us to calibrate accelerometer data which subsequently can be used to identify sub-lethal effects of disturbance such as higher energy expenditure (e.g. [18]).

4) Recent analysis highlights that human activity negatively impact distribution and alters behaviour of khulan in the Gobi [13, 30, 31]. There is also strong evidence that khulan avoid herders and livestock [9, 13], but the fact that khulan are regularly encountered by local people is often taken as evidence to the contrary. Whereas encounter rates of khulan with stationary infrastructure can be easily calculated based on tracking data, there is currently no way to track the movements of local pastoralists or their livestock in real-time at the landscape scale, and hence no possibility to calculate khulan-herder encounter rates. We expected camera collar images to provide insight into encounter rates and localities.

5) All equids are water dependent, meaning that they need regular access to drinking water, either in the form of open water or snow. In the south-eastern Gobi, khulan frequently access ground water by digging up to half a meter into dry riverbeds [32]. These diggings are small in dimension and invisible on open-source satellite imagery. What is more, water availability in many locations depends on the amount and temporal dynamics of precipitation in that particular year. Unfortunately, currently no comprehensive and verified map of seasonally or annually available surface water is available for the Gobi at a landscape scale. One way to detect available surface water is to use specific characteristics of khulan trajectories going to water (“*track-based waterpoints*”;[33]). Annual maps of such *track-based waterpoints* give a minimum estimate of waterpoints used in a given year. Many of these annual *track-based waterpoints* have been opportunistically confirmed during field work, but many more may have been missed or remain unconfirmed. We expected camera collars to provide a new way to ground-truth *track-based waterpoints*.

6) Environmental conditions in the Gobi are highly variable. Rainfall and snow are both sporadic and often highly localized. To understand khulan movements, tracking data needs to be closely matched with the environmental data at the time the animals used a specific location. For a species as far-ranging as khulan, the only way to match movements with climate data is to obtain remote sensing products, ideally available at a high frequency and a high resolution (e.g. MODIS NDVI or snow cover products [34]). Other important environmental variables like rain events must be modelled, e.g. by the Global Precipitation Climatology Centre for the *GPCC Daily first guess* product [35]. However, the coarse spatial resolution of the latter may not always reflect the local conditions which the animals are experiencing. We expected camera collars to measure agreement between data sources, and to assess how much local weather conditions differ from coarse-grained environmental data in a given year.

In the following, we report our experiences with a camera collar on an adult female khulan in the south-eastern Gobi of Mongolia focusing on the six issues detailed above. Based on our experiences with this one animal, we discuss the potential of the method for future applications on far-ranging species in this and other remote and highly variable ecosystems.

## Materials and methods

### Animal capture and collar details

We used a GPS‐Iridium collar with integrated camera (GPS PLUS-4 Collar, Vectronic Aerospace, Germany) and a pre-programmed drop-off mechanism (CR2a, Telonics, USA). Test deployment on a domestic horse showed that with a forward-facing camera, most images were obscured by the horse’s long lower jaw. Hence, we chose a side-facing camera and adapted the housing to provide the best fit for the anatomy of equids; the final collar weight was 1.8 kg (~1% of the body mass). Prior to deployment in the wild, we tested collar fit and its potential impact on animal wellbeing over several days on a domestic horse and subsequently over one year on a free-ranging Przewalski’s horse in the Gobi, which was observed by rangers on a weekly basis. We did not detect any collar-related injuries, substantial hair loss, discomfort or abnormal behaviours suggesting a negative impact (S1 File).

In October 2015, we equipped one adult female khulan in the south-eastern part of the Mongolian Gobi with the camera collar (for details on capture procedures see [36, 37]). Capture happened on non-protected public land and was authorized by the Mongolian Ministry of Nature, Environment and Tourism (capture permit 05/5656 issued 2015/09/17), the responsible entity for all protected species within and outside protected areas. The khulan was captured and collared within the context of the Core Biodiversity Monitoring requirement of the Oyu Tolgoi gold and copper mine. Capture and collaring was in accordance of best practice protocols for this species in the wild and the ethical commission at the University of Veterinary Medicine Vienna, Austria has been informed and given consent for the capture, handling and marking of khulan (ETK-15/03/2016).

When captured, the adult female (subsequently “collared khulan”) was accompanied by a foal and associated with a group of ~50 khulan. The camera collar remained on the collared khulan for exactly one year from capture to drop-date (16 October 2015–16 October 2016) and collected three sets of data: 1) GPS locations at hourly intervals (N = 8,787), 2) Photographs taken at 30-minute intervals before and after the full hour each day starting at 8:15 and ending at 19:45 (N = 8,719), and 3) Activity readings that consisted of accelerometer values averaged over 4.8-minute sampling intervals (N = 109,813).

**Fig 1:**
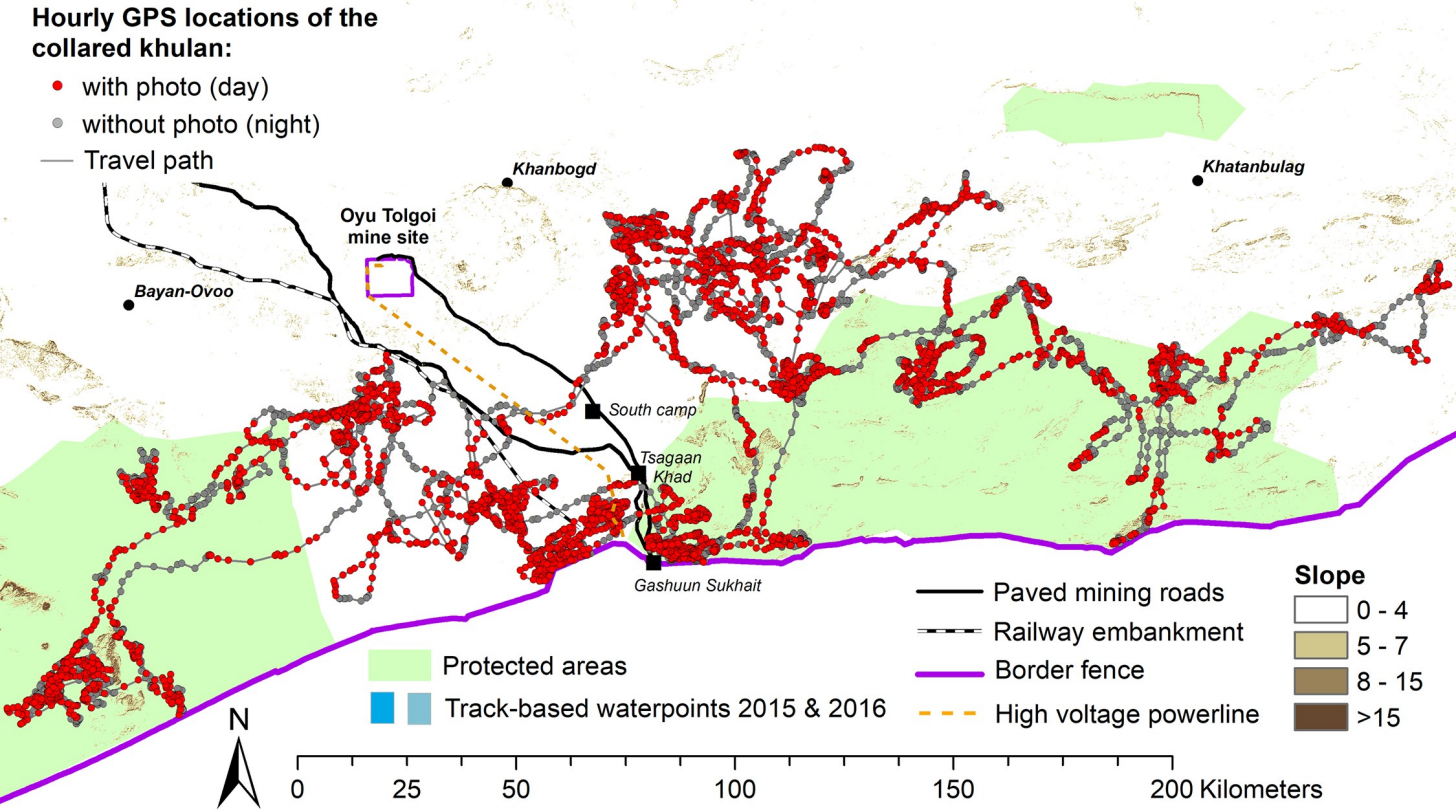
Study area in the south-eastern part of the Mongolian Gobi. Movement path with hourly GPS locations of the collared khulan from capture on 16 Oct 2015 to pre-programmed collar drop on 16 Oct 2016.

The total range of the collared khulan encompassed 22,198 km^2^ (100% Minimum Convex Polygon) and touched the fence along international border to China in the south, which constitutes an absolute barrier for khulan [31, 32]. The range is located around N42.700 / E107.600 and overlapped part A and B of the Small Gobi Strictly Protected Area (SPA) and the Tavan Tolgoi (TT) and Oyu Tolgoi (OT) mine infrastructure corridor. The latter includes two paved mining roads (“TT road” and “OT road”), a high voltage power line, the embankment for a railroad under construction (“railway embarkment”), and several service points (including the Tsagaan Khad coal re-loading facility and the Gashuun Sukhait border crossing point) (Fig 1).

The collared khulan used the western half of the roughly 100,000 km^2^ study area in south-eastern Mongolia that comprises the range of 41 different khulan that we have been radiotracking since August 2013 (see [13] for details). The study area is dominated by arid plains, interspersed with hills and low mountain ranges and elevations range from 683–1,884 m. The average annual temperature is around 7°C, but daily extremes vary between 40°C in summer and to −30°C in winter (Oyu Tolgoi weather station).

Average annual precipitation increases from the south-west to the north-east from an average 100 mm to around 150 mm. Surface water is available to wildlife at springs, many of which are only temporary and ephemeral pools in natural depressions fed by snow melt or rainwater. Khulan are also capable of accessing water by digging in dry riverbeds where the ground water table is high [32]. Although the availability of water at springs, pools, or diggings in dry riverbeds varies in time, their location is more or less spatially explicit; in other words, *when* water is available is more variable than *where* it will be available.

The location of waterpoints can be deduced from the specific characteristics of khulan trajectories going to water which are characterized by a succession of long, directed track segments to a waterpoint, followed by a narrow turn angle and long, directed track segments away from water (*track-based waterpoints*; [33]). Heavy rain showers can additionally result in standing water over large stretches of the Gobi for a short duration (normally hours, but sometimes days), basically bringing the water to the khulan, rather than necessitating khulan to go to water. The same is true in winters, when snow covers the ground, because khulan can eat snow as a water source [38, 39].

Vegetation is sparse and dominated by desert-steppe and semi-desert plant communities, particularly *Artemisia* spp., *Allium* spp., *Stipa* spp., and *Anabasis brevifolia*. There are a few tree species, including saxaul (*Haloxylon ammodendron*), elm (*Ulmus pumila*), and poplar trees (*Populus diversifolia*) which are confined to river valleys and large basins [40].

The khulan population in the south-eastern part of the Gobi is estimated to number >35,000 individuals [7]. Other large mammalian wildlife in the area include goitered gazelles (*Gazella subgutturosa*), and Mongolian gazelle (*Procapra gutturosa*) on the plains, and argali wild sheep (*Ovis ammon*) and ibex (*Capra sibirica*) in the mountains. Large mammalian carnivores include grey wolf (*Canis lupus*), Eurasian lynx (*Lynx lynx*), and snow leopard (*Panthera uncia*; for mammalian species see [41]).

The region is at the center of the cashmere goat industry in Mongolia, and livestock products generate the main income of local herders [6]. The rural populations consist of semi-nomadic livestock herders, keeping sheep and goats, horses, cattle and camels (in 2017, amounting to ~20,000 herding households with roughly 8,000,000 heads of livestock in the three Gobi provinces Umnogovi, Dundgovi, and Dornogovi; Mongolian Statistical Information Service 2017 at http://www.1212.mn/en/).

### Analysis

#### Image database

Over the 12 months deployment time, the camera collar collected 8,719 out of the 8,788 expected images (99% success rate). Of the images taken, 838 provided no information or only limited information because they were black (N = 510; image taken after sunset), blurry (N = 315; because of low light or animal movement), the lens was temporarily covered with mud (N = 6), only sky was visible (N = 4), or the image was white (N = 3); resulting in 7,881 (90.4%) images available for coding of khulan or habitat relevant information. An additional 8 black images provided information on infrastructure, due to the visibility of artificial lights.

Images were hand-coded by KS following a coding scheme aimed at determining: numbers of khulan, presence of foals, close association with other khulan, the behaviour of the animals visible in the images, horizon tilt of the image as a proxy for head position, proximity to herder camps, livestock, and infrastructure, habitat type, and weather (for the last two, with a special interest in the availability of water at waterpoints and/or in the form of rain or snow). For the coding scheme and coding examples, see S2 File.

Coding of images was very time-consuming and on average took 2.5 minutes for a total 325 hours for the entire image dataset (not counting double-coding or quality checking). Coding reliability and consistency was qualitatively assessed by parallel coding and cross-referencing of codes for the first 200 images between KS and PK, and for a random selection of 50 images between KS and JP.

#### Presence of khulan in the images

Each image was investigated for the presence of the khulan’s own body parts (“selfie”) or the shadow of the collared khulan and the presence of other khulan. We initially tried to identify the sex of other khulan in the image which proved extremely time consuming and rarely possible so that we gave up after the first 100 images. Foals were identified based on small body size and juvenile features in summer, and “fluffy-looking” fur and a shorter tail in winter to early summer (before the birth of a new cohort of foals). All khulan clearly visible in an image were counted and the background was investigated for additional, but uncountable khulan.

#### Behaviour

We classified the main behavioural categories of the collared khulan from all images that showed enough of the animal’s own body or shadow. We also classified the individual behaviour of other khulan in all images with ≤3 khulan and the group behaviour (shown by the majority of individuals) in all images with >3 khulan. Wherever possible we differentiated between: *Feeding*, *Standing*, *Lying down*, *Walking*, *Running*, and *Other*. For the group behaviour, we also used *Mixed* if multiple behavioural categories occurred at about equal frequency (Table in S2 File).

For diurnal trends over the daytime hours, we assigned the image taken at a quarter-to and a quarter-past to the respective full hour (e.g. images taken at 7:45 and 8:15 were assigned to 8:00).

#### Activity–image tilt & activity sensor to classify behaviour

**Fig 2:**
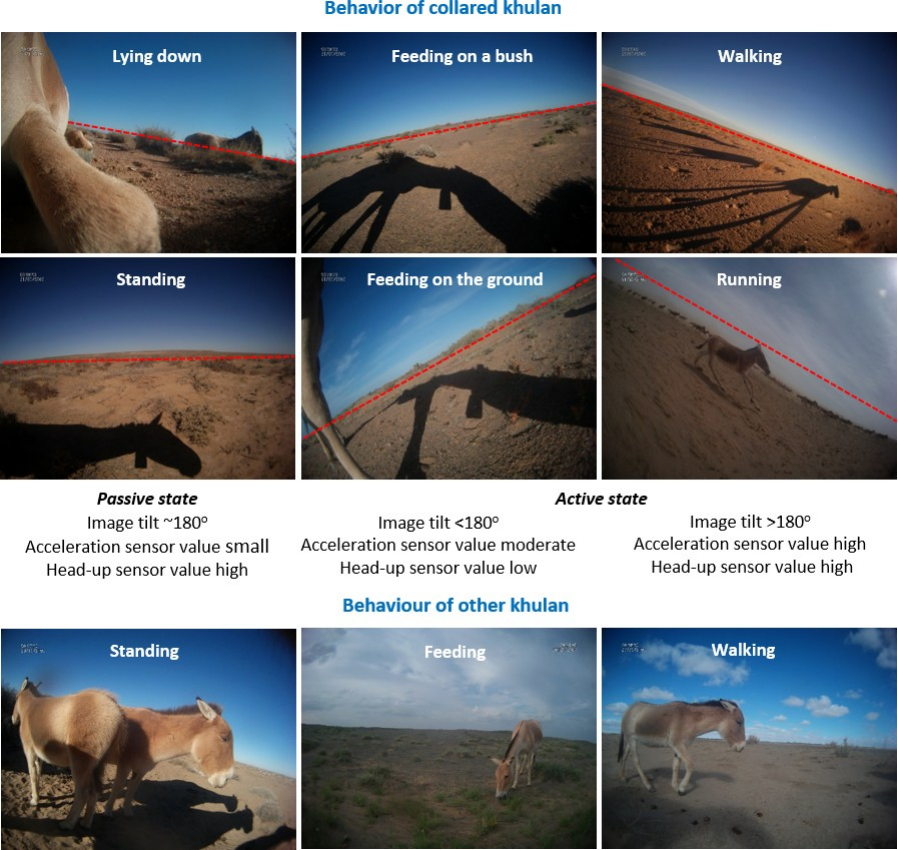
Khulan behaviour based on camera collar images. Top: Deducing behaviour of the collared khulan from body parts and the shadow visible in images and the expected values for Image tilt and activity sensor values. Bottom: Main behaviour of other khulan seen in the camera collar images.

The sideways orientation of the camera resulted in a tilted horizon of the image (“horizon tilt”) when the collared khulan lifted or lowered its head, and the horizon was close to horizontal when the khulan was standing (Fig 2). As the head position varies with different behaviours, we predicted that the horizon tilt could possibly be used as a proxy for behavioural categories–especially feeding–and hence measured the horizon tilt in each image using the open source software *ImageJ* (https://imagej.net/ImageJ). Tilt values were converted into degrees with 180^o^ representing a straight horizon, <180° a tilt up (right side of the horizon higher), and >180° a tilt down (right side lower) (variable: Tilt_degree).

The integrated 3-axis activity sensor of the collar measured the head angle and acceleration 4 times per second over a 4.8-minute sampling interval. The head-angle sensor is a proxy for posture. It measures the proportion the head exceeds a user-defined angle towards the vertical axis during the sampling interval. The acceleration sensor is a proxy for the overall activity status. It measures the proportion all three axes exceed a second user-defined threshold. Values for the two sensors range between 0 and 255, whereby 0 means that the user defined threshold was never exceeded and 255 means that the user-defined thresholds were exceeded for 100% of the interval. We used thresholds of 137 (representing 27°) for the head angle and 20 for acceleration based on observations of domestic horses fitted with the same brand of collars (Schirrmann unpubl. data 2008). Unfortunately, for this particular collar the head angle sensor was mounted upside down, thus measuring head-up rather than head-down values. The faulty orientation most likely results in a reduced performance to differentiate behaviour as head-up is not as typical for any particular behaviour as is head-down for feeding.

The signatures of the horizon tilt and activity sensors overlapped greatly between the two passive behavioural categories *Standing* and *Lying down* (S1 Fig). As we were primarily interested in discriminating between behaviours with different energy expenditure consequences (active versus passive behavioural status) or habitat requirements, we combined *Standing* and *Lying down* into *Resting* and contrasted them with the active mode *Feeding* (requiring pasture) and *Walking* (which may be less habitat-specific).

To each image we assigned horizon tilt and the head-up and acceleration values from the 4.8-minute activity interval immediately preceding and following each image (note that the horizon tilt measure reflects the instant of the photograph, whereas the head-up value of the activity sensor is an average value over the 4.8-minute sampling interval before or around the time the image was taken). In addition to acceleration (*Sens_Accel*) and head-up (*Sens_Head*.*up*) in the 4.8-minute sampling interval preceding the image, we also calculated the sum of both (*Sens_Sum*), and the absolute difference in the acceleration and head-up values between the two sampling interval bracketing the image time stamp (*ABS_Diff_Accel* and *ABS_Diff_Head*.*up*).

We used supervised classification trees to classify the three behavioural categories, using the images for which the behaviour of the collared khulan had been hand coded to train the algorithm (CART algorithm in R package *rpart*; [42]. As the classification tree algorithm needs about equal numbers of observations for classification, we randomly selected a subset of 150 observations for *Resting*, *Feeding* and all 116 observations for *Walking*. We subsequently used 2/3 of the observations to train the model and the remaining 1/3 to assess model fit. We pruned the classification tree, optimizing for the highest overall correct classification based on the confusion matrix (S3 File).

We subsequently classified the behaviour for all 8,719 images using the horizon tilt of the image and the automatically recorded activity sensor values (tilt & sensor model). For the 109,813 activity intervals we only used the automatically recorded acceleration and head-up sensor values (collar sensor only model). For diurnal trends over the daytime hours, we assigned the activity intervals to the full hour of the interval (e.g. intervals from 7:00:00 to 7:59:59 were assigned to 7:00). To visualize the activity readings on the map, we interpolated their position along the respective hourly trajectories, based on the difference of their timestamp from the hourly GPS fix.

#### Proximity to human activity and infrastructure

We checked all images for the presence of infrastructure, humans or livestock. For infrastructure, we double-checked all pictures with GPS locations <1000 m from focal infrastructure (the paved TT and OT mining roads, the railway embankment, the high voltage powerline, and the fence along the international border, see Fig 1) a second time, this time specifically looking for evidence of infrastructure.

#### Landscape types, with a special focus on waterpoints

We classified habitat type based on easily recognizable landscape features: *Plains*, *Hills*/*Mountains*, *Dry Riverbeds*, *Waterpoints (“image-based waterpoints”)*, and *Near waterpoints*. We were particularly interested in detecting new waterpoints and confirming *track-based waterpoints* which had been deduced from the trajectories of all GPS-satellite collared khulan over 12 months periods from 1 August to 31 July the following year. To make sure no water points were missed in the images, all images with locations at *track-based waterpoints* for 2015 and 2016 were checked a second time for evidence of being at or near a waterpoint.

To determine if *image-based waterpoints* were identical with *track-based waterpoints*, we buffered the GPS location of all *image-based waterpoint* with 1000 m (to account for possible khulan movements within the 15-min intervals around the full hour), merged those that were closer than 2 km apart to get unique *image-based waterpoints* and checked whether they intersected with the *track-based waterpoints*.

#### Climate, with a special focus on rain and snow

We coded weather conditions in the images as one of the following categories: *Clear*, *Cloudy*, *Raining*, S*nowing*, or *Sandstorm*.

For comparison of rain events in the images with regional climate data we used hourly precipitation records from the local weather station at the OT mine site (located at the northern extent of the collared kulan’s range at N43.00833 / E106.8430; Fig 1) and for comparison with global data we used the spatially-averaged estimates based on ground station data from the Global Precipitation Climatology Centre (GPCC) of the German Weather Service. The *GPCC First Guess Daily* product is provided on a relatively coarse, 0.5-degree grid (roughly 41km E-W x 55km N-S at that latitude) at: ftp://ftp.dwd.de/pub/data/gpcc/html/gpcc_firstguess_daily_doi_download.html. For analysis, we associated each of the hourly khulan track positions with the corresponding precipitation estimate from the GPCC product (S3 Fig).

After the first round of image coding, we checked the image on days with GPCC values >2 mm a second time, this time specifically looking for at least one image with evidence of recent rain events such as rain water on the ground, wet ground, or water of fog on the lens (see examples in Figure I in S2 File). We subsequently compared how well daily rain measured by the local weather station or the large scale GPCC product corresponded with evidence of rain events from the camera collar images.

We assessed snow cover from the camera collar images based on three categories: *None*, *<25%*, *25–50%*, and *>50%*. We subsequently compared days with images showing snow cover against daily estimates of average snow cover over the entire 94,000 km^2^ range used by collared khulan in the south-eastern Gobi, based on daily MODIS/Terra Snow Cover product (Daily L3 Global 500m Grid, Version 6 (MOD10A1) available at: http://nsidc.org/data/mod10a1; S3 Fig).

## Results

### Associations with other khulan

In 4,397 (56%) of the 7,881 images available for coding, other khulan were visible. It was not always possible to determine whether more khulan were in the background, but in 3,294 images (75%) we were confident about the absence of other khulan. Only 17 images had “many” uncountable khulan in the background and another 45 images had a “few more” khulan in the background. The number of other khulan countable in an image ranged from 1 to >80 (mean = 1.8, median = 1, mode = 0, SD = 3.3) with peak numbers seen in late spring and summer (>80 on 20 Aug 2016, >66 on 26 Jul 2016, >50 on 24 Jul 2016, and 53 on 16 May 2016). In 32 images, 20 or more khulan were visible and in an additional 10 images “many” more khulan were visible in the background but could not be reliably counted. The combined 42 images (0.5%) with larger khulan aggregations were primarily located throughout the central part of the collared khulan’s range (Figure in S4 File).

**Fig 3:**
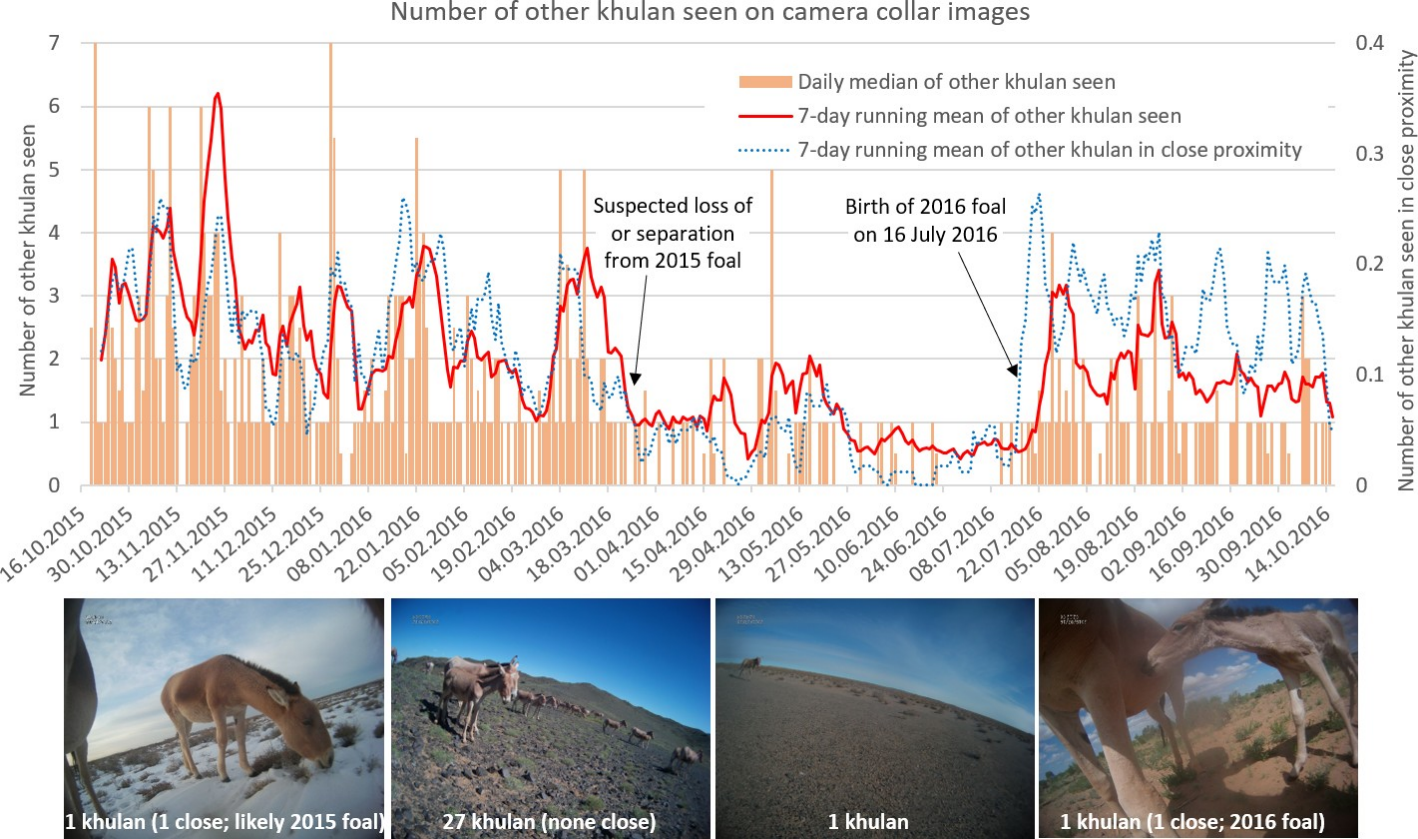
Association of the collared khulan with other khulan based on camera collar images.

Over the year, the association of the collared khulan with other khulan showed a clear seasonality with a higher occurrence of larger aggregations in winter (late October–March), a low during early summer (April–mid July), and intermediate aggregation sizes in summer and early fall (August to mid-October). A frequent switch between days with and without images of other khulan primarily occurred from the end of March until mid-July, including a 2-week “solitary phase” in early July (Fig 3; Table in S4 File). Images from 16 Jul 2016 showed a newborn foal in close proximity being suckled and groomed, confirming a reproduction event (Fig 3).

In 765 (9.7%) images other khulan stood close to the collared mare; 678 images showed one khulan in close proximity, 69 images two khulan, 16 images 3 khulan, and only 2 images 4 khulan. As foals tend to stay close to their dams, the trend in *other khulan in close proximity* seems a good proxy for the presence and loss of or separation from a foal (Fig 3).

### Khulan behaviour

A total of 2,779 (35%) images showed parts of the collared khulan’s shadow (N = 1,918) or significant portions of her body (N = 1,317); of those, 1,630 (12%) showed details that enabled us to classify her behaviour. Additionally, we were able to classify individual behaviour for ≤3 other khulan in 2,981 images (totaling 4,901 individual behavioural records) as well as the main group behaviour from an additional 1,249 images that showed >3 khulan (with 1,069 images showing 4–10 khulan, 152 images showing 11–20 khulan, 19 images showing 21–30 khulan, and 9 images showing >30 khulan).

The collared khulan spent most time *Feeding*, followed in decreasing order of frequency by *Standing*, *Lying down*, *Walking*, and in a few rare cases *Running* or *Other*. Her overall time budget was similar to the time budget of other khulan seen in the images (Table 1).

**Table 1:** *Main khulan behavioural categories based on different cohorts of khulan seen in the images during daylight hours from 16 Oct 2015 to 16 Oct 2016*.

Source	% Behavior							N	
	Feeding	Standing	Lying	Walking	Running	Other	Mixed	Images	Individuals
Collared khulan	52.4	30.2	9.4	7.1	0.4	0.5	NA	1,630	1
≤3 khulan*	53.3	30.8	5.2	10.3	0.3	NA	NA	2,981	≤4,901
Group behaviour*	54.6	30.3	0.6	9.8	1	NA	3.8	1,249	NA

*These are mutually exclusive–group behaviour was determined if >3 khulan were clearly visible

### Behaviour of collared mare, and the ability of sensors to identify behaviour

For the four main behavioural categories *Feeding*, *Resting*, and *Walking*, a total of 1,616 images (20.5%) were available that showed the collared mare and additionally had values for the horizon tilt and the activity sensors (measuring head-up and acceleration).

Classification tree analysis was able to correctly identify *Feeding* (93%) based on horizon tilt and *Resting* (84%) based on the acceleration sensor value but performed rather poorly for *Walking* (59%), which was mainly confused with *Feeding*. Without the horizon tilt of the image, the classification tree analysis using the sensor value for acceleration and head-up performed not as well for *Feeding* (75%) and *Resting* (68%) but improved for *Walking* (65%; for details see Figure and Table A in S3 File).

**Fig 4:**
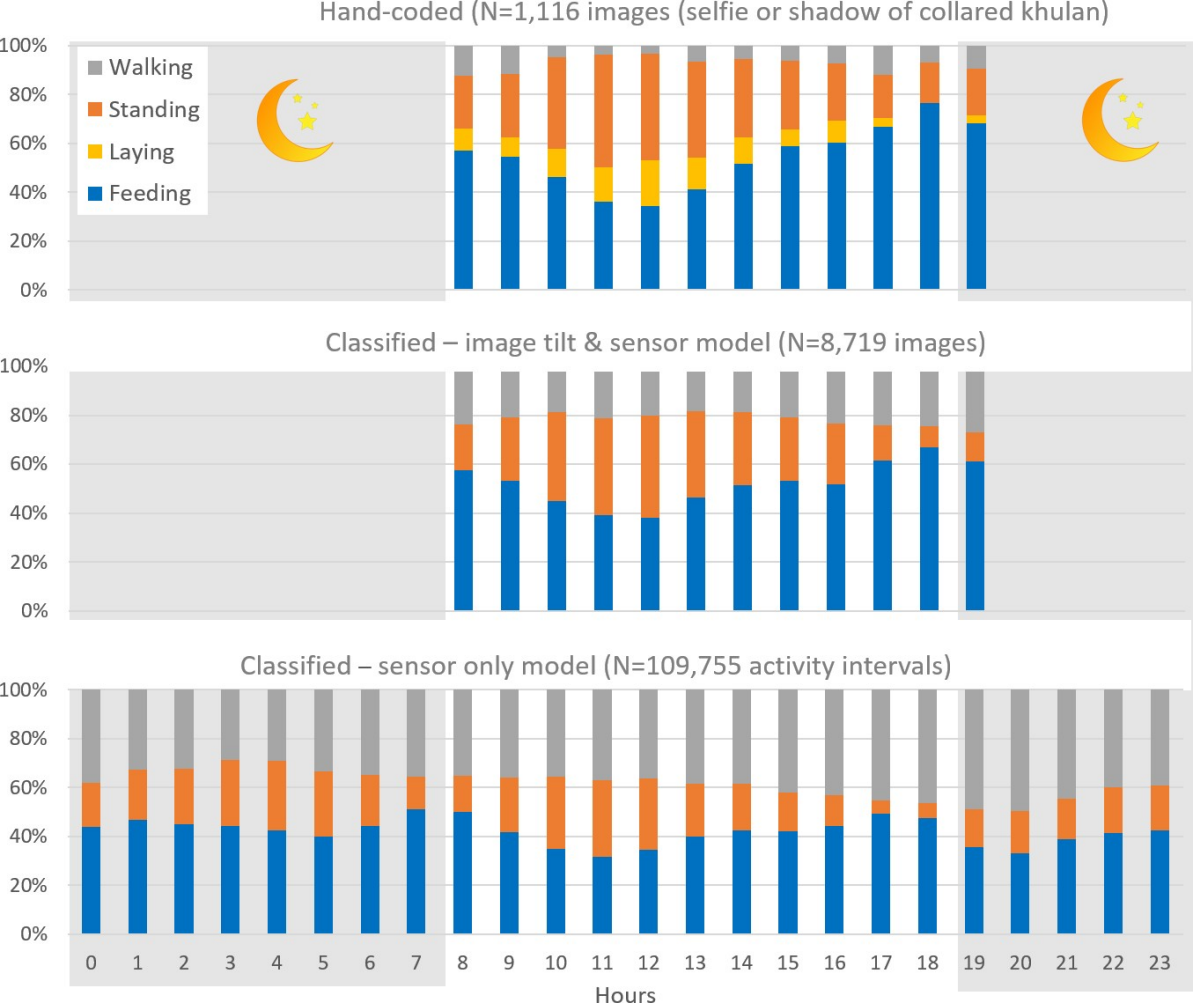
Diurnal pattern of main behavioural categories for the collared khulan. White background = hours with daylight throughout the year.

The overall diurnal pattern of the behaviour of the collared khulan remains the same regardless of whether it was based on hand-coded images, classified images using the tilt & sensor model, or classified using all activity intervals. However, the relative proportions change with *Feeding* and *Resting* becoming under- and *Walking* overrepresented in the classified dataset, especially when using the sensor-only classification model (Fig 4; Table B in S4 File). However, in part, this is likely the result of the faulty orientation of the sensor measuring head-up rather than head-down.

**Fig 5:**
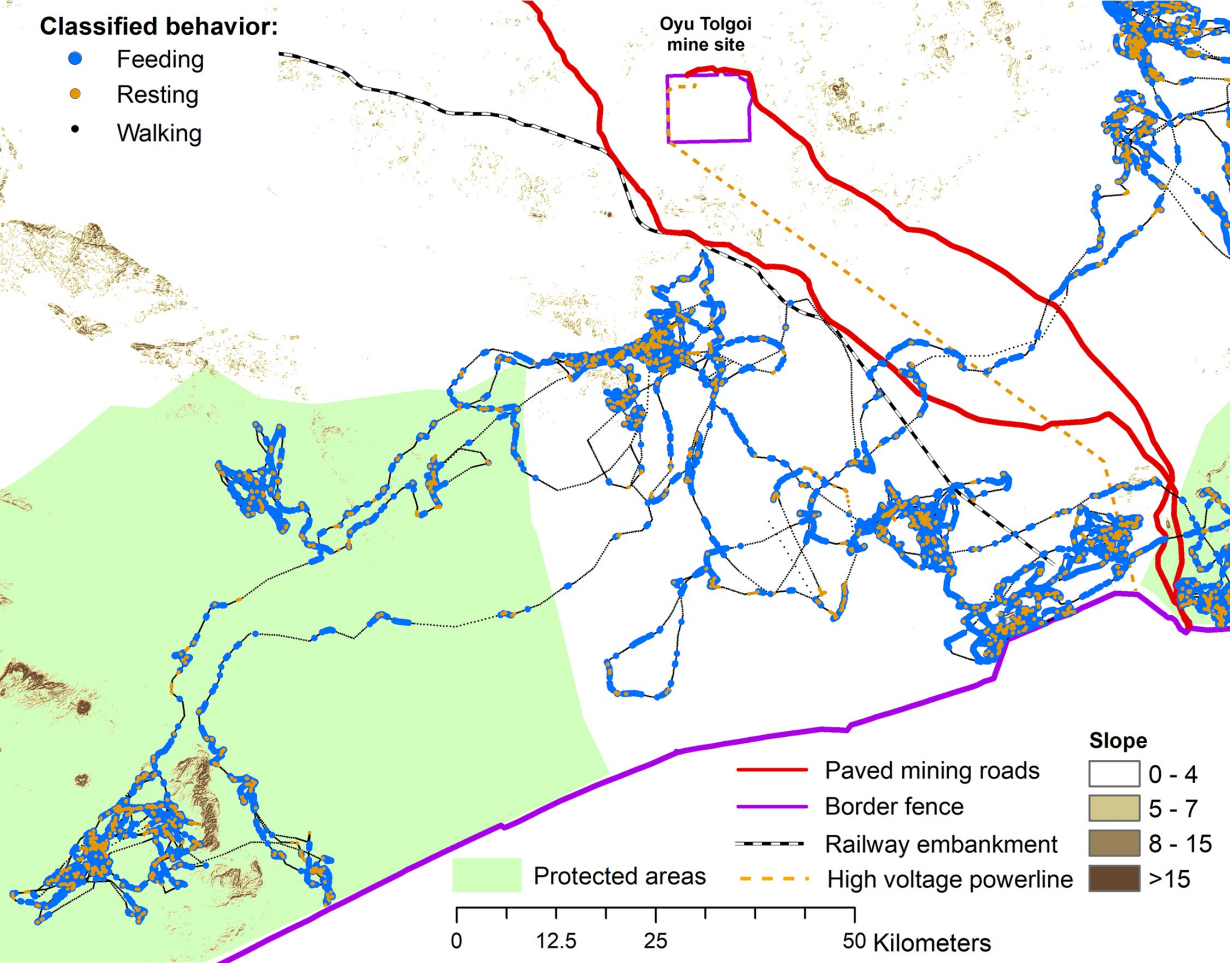
Interpolated locations of 4.8-minute activity intervals of a collared khulan. *The map exemplarily shows the trajectories of the western part of the collared khulan’s range*. *Trajectories with a mix of* Feeding, Resting, *and* Walking *intervals are at times interrupted by almost pure* Walking *intervals*.

A first visual inspection of the spatial distribution of *Feeding*, *Resting* and *Walking* intervals showed that *Resting* is interspersed with *Feeding and* suggests that these two behaviours may not differ in respect to habitat requirements. *Walking* is intermixed with both *Feeding* and *Resting*, but at times occurs exclusively and seems to characterize long-distance displacements from one area to another. These long-distance displacements may characterize areas of low pasture value or specific events (e.g. flight or search behaviour; Fig 5).

### Proximity to livestock and infrastructure

Proximity of khulan to livestock was rarely recorded, with only 30 images (0.4%) showing livestock: 23 showing Bactrian camels, 4 horses, 2 cattle and 1 goats. No images showed people, herder camps, or vehicles (other than on the paved TT or OT road).

**Fig 6:**
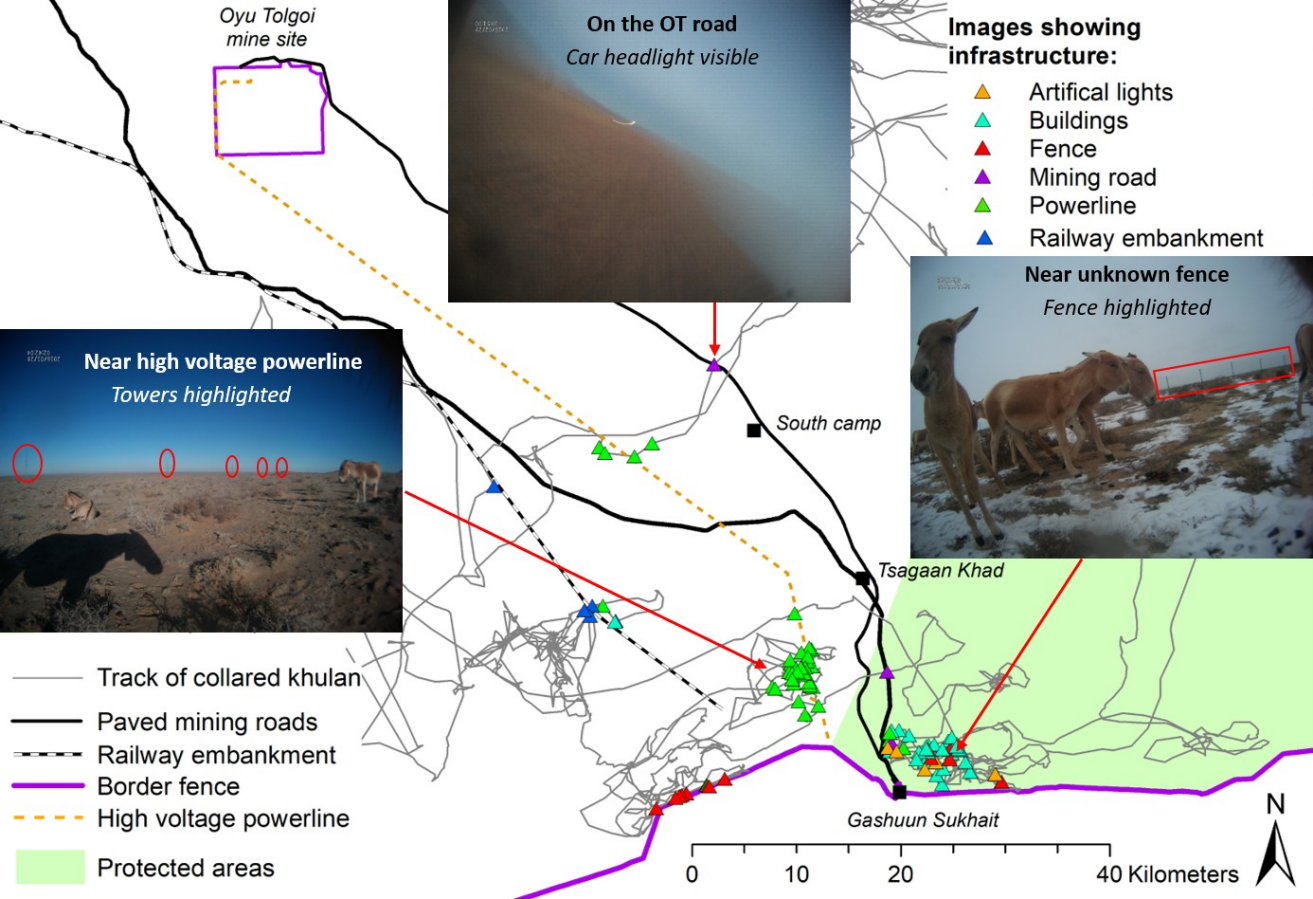
Location of 131 images where infrastructure was visible. Red arrows point towards infrastructure complexes which we had not been aware of prior to seeing the camera collar images.

Infrastructure was visible in a total of 131 images (1.6%) from 35 different days and at 106 different locations. In decreasing order of frequency, the images showed: the high voltage powerline, buildings, fences, the OT or TT mining roads, and the railway embankment (Fig 6; S1 Table).

All infrastructure images were from fall and winter (October 2015—February 2016), when the collared khulan stayed near the TT and OT mine infrastructure corridor. The images also identified infrastructure previously unknown to us: buildings and a fence in the south-western part of Small Gobi B SPA (Fig 6) which are not part of the Gashuun Sukhait border crossing, a service camp near the railway embankment, and one service camp at the southern end the OT road.

In the 131 images showing infrastructure, 33 images also show the behaviour of the collared khulan and an additional 80 show the behaviour of other khulan. Images showing the powerline, buildings, fences, and the railway embankment showed khulan feeding and resting (including lying down). Images of the mining roads are rare, and mostly from the dark hours; they showed only one other khulan which was feeding (Table 2).

**Table 2:** Khulan behaviour in images with infrastructure.

Infrastructure type	N	N images showing		Behaviour of khulan in images^1^				
		collared khulan	other khulan	Lying	Standing	Feeding	Walking	Mixed
Powerline	66	23	40	5	18	36	10	0
Buildings	40^2^	6	25	6	33	69	24	1
Fences	14	3	11	0	4	10	6	0
Mining roads	6^3^	0	1	0	0	1	0	0
Railway embankment	5	1	3	0	3	3	0	0

^1^Represents the sum of: Behavior of collared khulan and other khulan seen (1–3 khulan and main group behaviour)

^2^Five images near border where black, but showed artificial lights

^3^Four images were black, but showed artificial lights; one was blurry (Fig 6).

### Ground truthing of remote sensing or modelled water resources

#### Spatially explicit waterpoints

**Fig 7:**
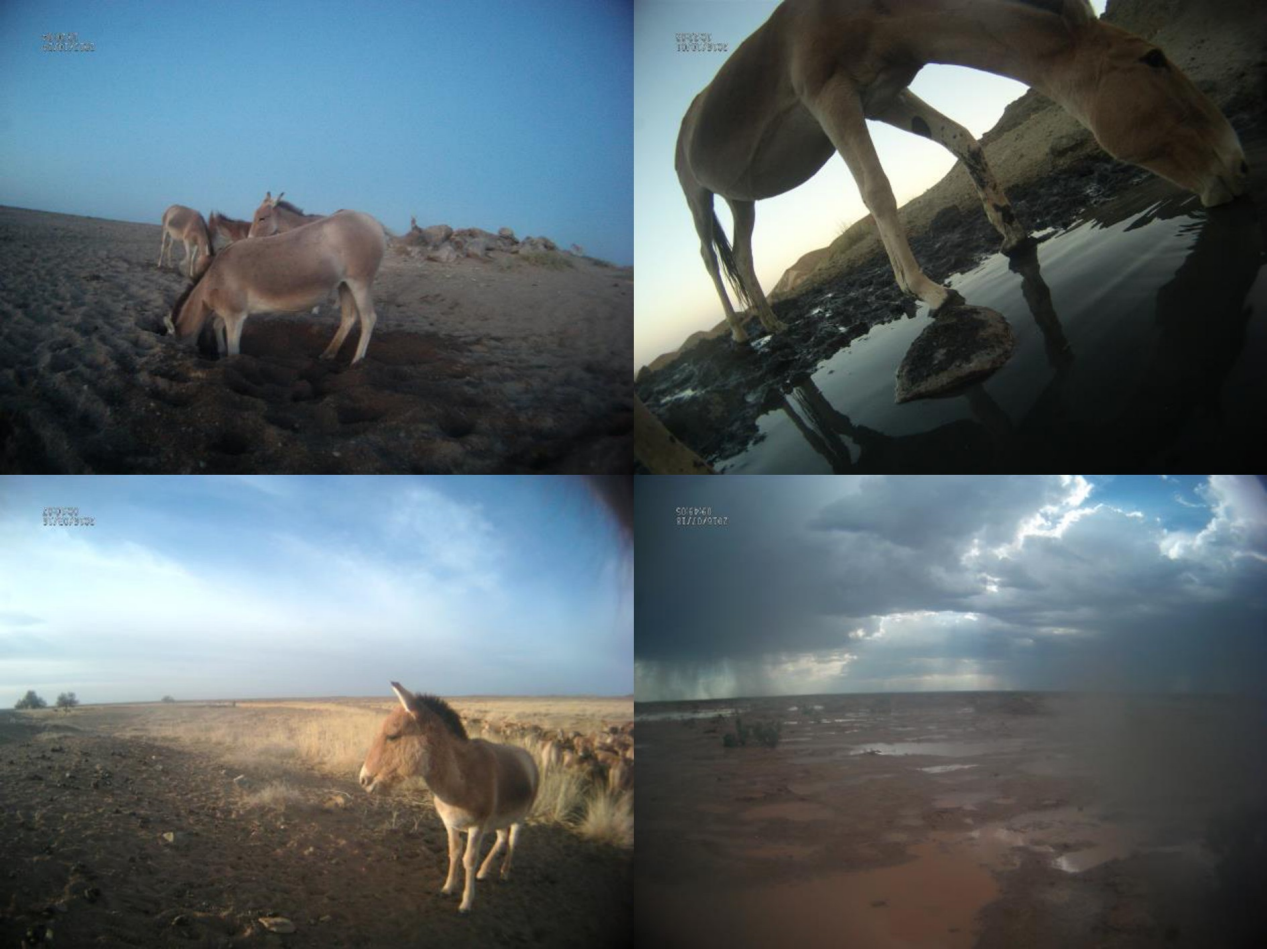
Water sources available to khulan. From top left to bottom right: i) Waterpoints: Khulan digging for ground water in a dry riverbed; Khulan drinking from a pool; Waterpoint deduced from vegetation, tracks, and animal behaviour; ii) Short term water available over larger areas: Rainwater over larger part of the ground from ongoing rain event.

Landscape type was recorded for 7,838 images (99%), with by far the most frequent being *Plains* (S2 Table). *Waterpoints* (diggings in dry riverbeds and single, clearly delineated open water bodies (“pools”) were only visible in 59 images (0.7%) at 28 different locations (>2 km apart; Fig 7). Sixteen of these 28 locations (57%) confirmed waterpoints of our *track-based waterpoint* layer for 2015 or 2016 and 12 identified additional waterpoints (2 of which had been identified by our *track-based waterpoint* layer from earlier years).

#### Short term water available over larger areas due to precipitation

**Fig 8:**
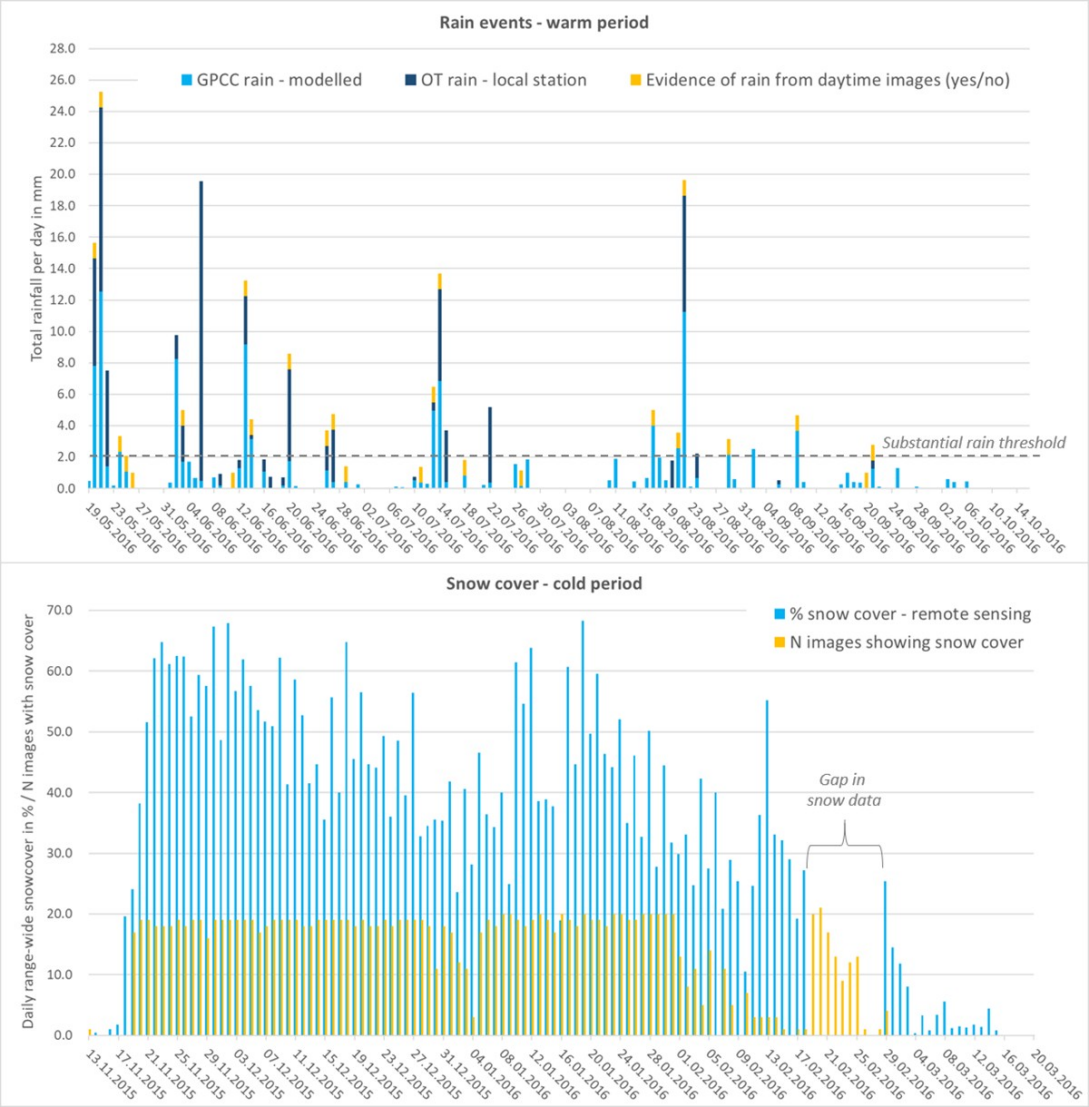
Match between different daily precipitation data and camera collar image information. Top: Match between % snow cover of the 100,000 km^2^ khulan range from MODIS/Terra daily snow cover product and the number of images from camera collar showing snow cover. Bottom: Match between rain events derived from the GPCC First Guess daily product and measured at a local weather station near the Oyu Tolgoi (OT) mine and evidence of rain in at least one image from the camera collar on the same data.

Weather conditions were coded from 7,856 images (99.7%). In most images, the sky was clear (66%) or cloudy (32%); sandstorms (1.1%), rainfall (0.8%), and snowfall (0.1%) events were rarely captured (Table in S5 File). On 25 days during the non-snow season we found evidence of recent rainfall (Fig 7-bottom right, Fig 8). The images confirmed rain on 12 of 14 days for which the GPCC data predicted substantial rain (≥2mm) and for 9 of 12 days on which the Oyu Tolgoi weather station recorded substantial rain. The camera collar images recorded rain on 3 days when no rain was predicted by the GPCC or recorded by the OT weather station. However, only days with substantial rain had been double-checked for evidence of rain events.

Actual snowfall events were rarely captured on the images, but the resulting snow covering the ground was visible in 1,550 images (19.6%) from mid-November 2015 until mid-February 2016. The amount of snow on the ground also allowed some insight into the timing of snow events (Figure in S5 File). A mismatch between the satellite-derived snow cover estimate on the landscape scale and the local snow conditions based on the camera collar images occurred mainly in late winter and/or when the snow cover on the landscape scale was <25% (Fig 8; Figure in S5 File).

## Discussion

### Coding khulan behaviour from camera collar images

The overall aim of this analysis was to explore the potential of camera collars for addressing the six key questions raised in the introduction. Because analysis was restricted to one animal, the results were kept descriptive as they do not allow to draw wider conclusions about the khulan population in the south-eastern Gobi, but rather show how the image information can help to broaden our understanding of a far-ranging ungulate in such a remote and highly variable ecosystem. We are also fully aware that the precision of the behavioural classification for this collar was compromised by the faulty orientation of the head-down sensor but given the exploratory goal of our analysis we feel this is of minor importance. We also want to point out that currently available camera collars do not have night vision technology and hence can only provide insight into daytime behaviour and habitat use.

A qualitative comparison of individual coders suggested good agreement (counts of other khulan usually agreed within 1 or 2 animals, and behavioural classifications were consistent) and a high self-consistency of the main coder (KS). However, coders differed substantially in their assessment of which animals were foals as with increasing age foals started to look more and more like adults. But because foals tend to stay close to their dams, the number of *other khulan in close proximity* seems a more objective index for detecting the birth or loss of a foal.

The wide-angle lens of the camera resulted in distant features becoming rather small and detecting infrastructure and other landscape details in the distance required zooming in and out of the image. Double-coding and double-checking for known infrastructure suggested that up to 25% of infrastructure may be missed during a single coding round.

Coding was extremely time-consuming so that for image analysis to become an integral part of analysis, some form of automated pattern recognition will have to be used (i.e. artificial intelligence techniques [43]). The hand-coded dataset obtained during this study, can now be used as a training dataset for automated image recognition.

### Identifying reproductive events

The collar camera images provided important data about the time and location of foal birth; basic ecological information that is largely missing and difficult to collect in the field. Images further allowed inferences about how long a mare was accompanied by a foal. While the birth of the foal 2016 could be pinpointed to the day by the camera collar images, the fate of the foal 2015 remains unknown. The sudden decline in the number of images with *other khulan in close proximity* and the increase in images without any other khulan strongly suggests that the collared khulan either lost or was separated from her 2015 foal by late March 2016.

The collared khulan’s movements during the first four days post-parturition were spatially confined (13.9 km^2^) and the overall time budget based on the activity intervals was shifted towards a high proportion of walking (64.7%) at the cost of feeding (13.2%; S3 Fig). If this pattern can be confirmed in other camera-collared adult females, it may be possible to infer reproduction events from the combined information of activity sensor data, and movement characteristics in khulan equipped with regular GPS-satellite collars as has been done for caribou (*Rangifer tarandus caribou*) [44].

### Identifying social grouping

Grouping patterns varied throughout the year, with aggregation lows in summer and aggregation highs in winter. The patterns observed on a day-to-day basis support the fission-fusion dynamics that have been hypothesized for khulan, as well as the overall seasonal grouping patterns based on line transects that have been previously reported from the south-western Gobi [45]. The information is relevant for planning population surveys, which ideally would be timed to coincide with times when the population is the least aggregated and the variation in group sizes is lowest [7, 46].

Furthermore, repeated seasonal aggregations in certain areas may help to identify priority areas for conservation and can be cross-referenced with areas of high use based on GPS tracking data alone. Daily or regional aggregation indexes also have the potential to be used as covariates or weights in resource selection functions [47]. By deploying camera collars, it should also be possible to detect whether large aggregations are caused by barriers [48], as such barriers would likely be visible on the images, as was the case for fences in the khulan example.

### Khulan behaviour

The images provided a large pool of instantaneous scan samples [49] of the focal individual–the collared khulan–and other khulan that the focal individual was associated with. This pool allowed us to calculate basic time budgets, which can be broken down over the daytime hours or by month or season. What was more, those images depicting body parts or the shadow of the collared khulan allowed us to train the activity sensor data and to subsequently classify the behaviour for all image locations and all activity intervals.

The precision of the behavioural classification in this particular collar was compromised by the faulty orientation of the head-down sensor. But more generally, the rather long 4.8-min recoding intervals of the activity sensors only allowed to classify very basic categories. Furthermore, the category *Running* and *Other* occurred so rarely, that there was not enough data to train the classification algorithm for a category *Other*. Hence, *Running* and any other behaviour which is associated with increased body movements (e.g. social interactions, self-grooming etc.) are most likely classified as *Walking*, which may explain why the proportion of *Walking* increased in time budgets calculated based on the activity sensor alone.

Khulan tend to walk and feed in a stop-and-go fashion, and regularly lift their heads to look around or make larger uninterrupted movements to the next forage patch. Consequently, distinguishing between walking or standing, standing or feeding, and walking or feeding from values averaged over 4.8-minute intervals must be expected to include substantial noise, as has been shown for other ungulates [50]. Using camera collars with video in combination with raw acceleration data can be expected to improve the ability to also detect short, but costly behaviour like vigilance [18].

The inferred behavioural information can be linked to the animal trajectories to investigate if and how certain landscape features or anthropogenic activities influence behaviour [18, 30, 51]. Sensor data from the collared khulan showed that while *Resting* and *Feeding* wre interspersed with short bouts of *Walking*, long bouts of pure long-distance *Walking* occur as well. This suggests that any disturbance or need that forces khulan to move long distances interrupts both feeding and resting, which is probably associated with increased metabolic costs.

### Proximity to livestock and infrastructure

The camera collar images provided new or additional evidence about behaviour near and avoidance of human activity. The lack of images of herder camps and the very small number of images with livestock adds to other circumstantial evidence suggesting that khulan avoid herder camps and their associated livestock [9, 13]. This information is extremely important as currently we have no possibility to map herder camps and livestock herds at the landscape level.

Mining-related infrastructure, on the other hand, were frequently encountered when the collared khulan moved in the mining infrastructure corridor. Multiple images of khulan close to the powerline and the railway embankment showed khulan resting or grazing, and do not immediately suggest strong avoidance behaviour. Images of the paved mining roads, on the other hand, were rare and limited to the low-light hours, which is circumstantial evidence that the collared khulan avoided crossing roads during daytime, when traffic is highest. The camera collar images also provided us with information about previously unknown infrastructure. However, unknown infrastructure which is totally avoided will not be visible on images, nor will those structures which are only avoided during daytime!

### Water availability for khulan

The camera collar images helped to confirm the location of waterpoints inferred from khulan trajectories as well as detect new waterpoints. However, our telemetry data of 41 khulan monitored between 2013–2017, suggests that khulan in the southern Gobi primarily drink at dawn and dusk and do not stay long at waterpoints. With the current technical set-up (images restricted to daytime hours at 30min sampling intervals and the camera being orientated to one side only), one can only confirm events or locations, but not reject them (e.g. it is not possible to calculate drinking frequency or to identify false positive waterpoints inferred from khulan trajectories).

The camera images also confirmed predicted rain events modelled from a network of global weather stations and landscape scale snow cover values. This is good news for the use of these values as model predictors. A certain mismatch in the global or landscape scale predictions for rain events and average snow cover suggest that remote sensing products may at times be too coarse to fully describe local conditions, hence somewhat reducing the predictive power of movement models.

### Public relation potential

Remote camera imagery, including still images and video footage from animal born systems has a huge media potential, as documented by an increasing number of images used in popular science articles and books (e.g. [52]) or documentaries like the National Geographic’s “Crittercam Chronicles” (http://natgeotv.com/me/crittercam-chronicles/about). These images and videos provide insight into the secret lives of animals and are almost guaranteed to attract public attention. Camera collar images are therefore an important added value when communicating new research findings to the public, developing attractive education material, and raising awareness for conservation issues. We used a small subset of the images from the khulan camera collar to supplement this publication with a popular version in a StoryMap format [53] at: https://arcg.is/1jP4L1.

### Conclusion

The camera collar we deployed allowed added insight into all six research complexes outlined in the introduction and has a huge potential for public relation work. In summary we argue that camera collars constitute a very valuable supplementary tool in wildlife tracking studies, especially in settings where there is limited scope for ground work due to large ranges and unpredictable movements of the study species, ecosystems prone to change, and remote areas. Additional aspects concerning food choice, habitat type, and pasture quality assessment could be additionally explored, but were beyond the scope of this study (Table 3).

**Table 3:** Overview of the added value of additional camera collars for ecological research on far-ranging species in remote and highly variable ecosystems, as exemplified by our khulan camera collar dataset.

GPS satellite tags	3-axis accelerometer	Remote sensing data
***Data generated***		
• GPS locations • Movement trajectories • Interaction with known landscape features and impacts • Interaction with other tagged animals	• Raw or averaged sensor data as a result of body posture and body movement	• Habitat types • Climate data • Productivity
***Added value from still or video or imagery***		
• Life-history events • Interaction with other untagged animals • Interaction with variable elements of the environment • Added information to put locations or sensor values into context	• Ground-truthing of sensor values against the known behaviour of the tagged animal, to train classification algorithms	• Ground-truthing of habitat types & climate data • Detection or confirmation of small, but potentially crucial habitat features or resources • Detection of local climate events
***Value in highly variable*, *remote ecosystems—Khulan example***		
• Foal birth data & presence of foal over monitoring period; potential to use trajectory characteristics to infer foaling in khulan with regular GPS satellite collars • Potential to better understand variables influencing fission-fusion social dynamics • Potential to incorporate the large variations in association with other khulan as a covariate in habitat suitability analysis • Potential to infer avoidance of disturbance from the frequency of interactions with known, but unmapped anthropogenic activities (e.g. livestock herding) *Not explored*: *Potential to identify food species consumed by other khulan*	• Body parts and shadow of collared khulan allowed for obtaining training data to classify main behavioural categories (Resting, Feeding, Walking) • Horizon tilt of image improved behavioural classification • Possibility to cross-reference the behaviour of the tagged animal with behaviour of untagged animals • Possibility to document behaviour at times or at locations of interest • Potential to use sensor data in combination with trajectory characteristics to infer life history events	• Confirmation of waterpoints inferred from trajectories of other khulan • Detection of new waterpoints • Detection of unknown infrastructure • *Not explored*: *Potential to ground -truth habitat types derived from remote sensing* • *Not explored*: *Potential to obtain data on plant species composition and % cover in a habitat with high intra- and interannual variability*
***Constraints of the system deployed***		
• Deployment time currently limited to about 1 year, due to weight and data storage constraints • Still images were restricted to daytime • While the high resolution of the still images allows for detailed inspection, it misses the dynamic aspect of video footage; sound may also provide additional information on behaviour or interaction between animals • High effort required for hand-coding of images • Higher costs of currently ~1.5 times the cost of a regular GPS-satellite collars	• The side-facing camera optimized the view of interactions with the environment and other untagged animals, but was less suitable to monitor behaviour and unsuitable to document food choice of the tagged khulan itself • Still images represent an instant and are hence suboptimal for calibrating sensor values averaged over a 4.8-minute interval • The 4.8-minute interval is too long to allow fine-scale behavioural differentiation	• No information about unknown features or habitats which are avoided

## Supporting information

S1 Table Infrastructure. (DOCX)

S2 Table Landscape type. (DOCX)

S1 File Collar testing prior to deployment on the khulan. (DOCX)

S2 File Coding instructions and illustrations. (DOCX)

S3 File Classification tree. (DOCX)

S4 File Other khulan seen. (DOCX)

S5 File Weather conditions. (DOCX)

S1 Fig Image tilt and activity sensor values of main behavioural categories. (DOCX)

S2 Fig Climatic conditions October 2015—October 2016. (DOCX)

S3 Fig Movements and behavior around foaling. (DOCX)
