# Supplementary material for: Through the eye of a Gobi khulan – Application of camera collars for ecological research of far-ranging species in remote and highly variable ecosystems
Source: PLoS One. 2019 Jun 4;14(6):e0217772. doi: 10.1371/journal.pone.0217772 (PMC6548383; doi:10.1371/journal.pone.0217772)
Supplement: S1 Table — (DOCX) [file pone.0217772.s001.docx]

## S1 Table. Infrastructure.

***S1 Table****. Infrastructure detected in the 1^st^ and 2^nd^ coding round.*

| **Infrastructure visible** | **1^st^ coding** | | | | | | **2^nd^ coding (all)** | **Total** |
| --- | --- | --- | --- | --- | --- | --- | --- | --- |
|  | **2015** | | |  | **2016** | |  |  |
|  | **10** | **11** | **12** |  | **1** | **2** |  |  |
| ***Mining road related**** |  |  |  |  |  |  |  | 6 |
| Artificial lights (coal road?) |  |  | 1 |  |  |  |  |  |
| Artificial lights (on TT road) |  |  | 1 |  |  |  |  |  |
| Artificial lights (on OT road) |  |  |  |  |  | 1 |  |  |
| Artificial lights near border (TT road?) |  |  | 1 |  |  |  |  |  |
| Trucks (on TT road) |  |  | 2 |  |  |  |  |  |
| ***Building / camps related**** |  |  |  |  |  |  |  |  |
| Artificial lights near border (fenced area with buildings) |  |  | 2 |  |  |  |  | 40 |
| Artificial lights near border (nothing nearby) |  |  | 3 |  |  |  |  |  |
| Buildings (near Gashuun Sukhait) |  |  | 30 |  |  |  |  |  |
| Buildings & fence (near Gashuun Sukhait) |  |  | 2 |  |  |  |  |  |
| Other (construction camp) |  |  |  |  |  | 1 | 2 |  |
| ***Fence related*** |  |  |  |  |  |  |  |  |
| Fence (along international border) |  |  | 1 |  | 5 |  | 3 | 14 |
| Fence (unknown origin) |  |  | 5 |  |  |  |  |  |
| ***Other linear structures*** |  |  |  |  |  |  |  |  |
| Powerline* |  | 1 | 6 |  | 47 | 4 | 8 | 66 |
| Railway embarkment & bridge* |  |  |  |  |  | 1 | 4 | 5 |
| **Sum** | **1** | **3** | **55** |  | **52** | **7** | **17** | **131** |
