# Supplementary material for: Through the eye of a Gobi khulan – Application of camera collars for ecological research of far-ranging species in remote and highly variable ecosystems
Source: PLoS One. 2019 Jun 4;14(6):e0217772. doi: 10.1371/journal.pone.0217772 (PMC6548383; doi:10.1371/journal.pone.0217772)
Supplement: S2 Table — (DOCX) [file pone.0217772.s002.docx]

## S2 Table. Landscape type.

***S2 Table****. Landscape types by season.*

| **Landscape type** | **Season** | | | | **Sum** |
| --- | --- | --- | --- | --- | --- |
|  | **Fall** | **Winter** | **Spring** | **Summer** |  |
| Plains | 1,684 | 1,552 | 1,741 | 1,966 | 6,943 |
| Hills & Small mountains | 125 | 215 | 306 | 160 | 806 |
| Dry riverbed | 6 | 2 | 4 | 1 | 13 |
| At waterpoint | 14 | 1 | 9 | 35 | 59 |
| Possibly waterpoint nearby |  |  | 13 | 4 | 17 |
| **Total** | **1,829** | **1,770** | **2,073** | **2,166** | 7,838 |
