# Supplementary material for: Through the eye of a Gobi khulan – Application of camera collars for ecological research of far-ranging species in remote and highly variable ecosystems
Source: PLoS One. 2019 Jun 4;14(6):e0217772. doi: 10.1371/journal.pone.0217772 (PMC6548383; doi:10.1371/journal.pone.0217772)
Supplement: S1 File — (DOCX) [file pone.0217772.s003.docx]

## S1 File. Collar testing prior to deployment on the khulan.

**
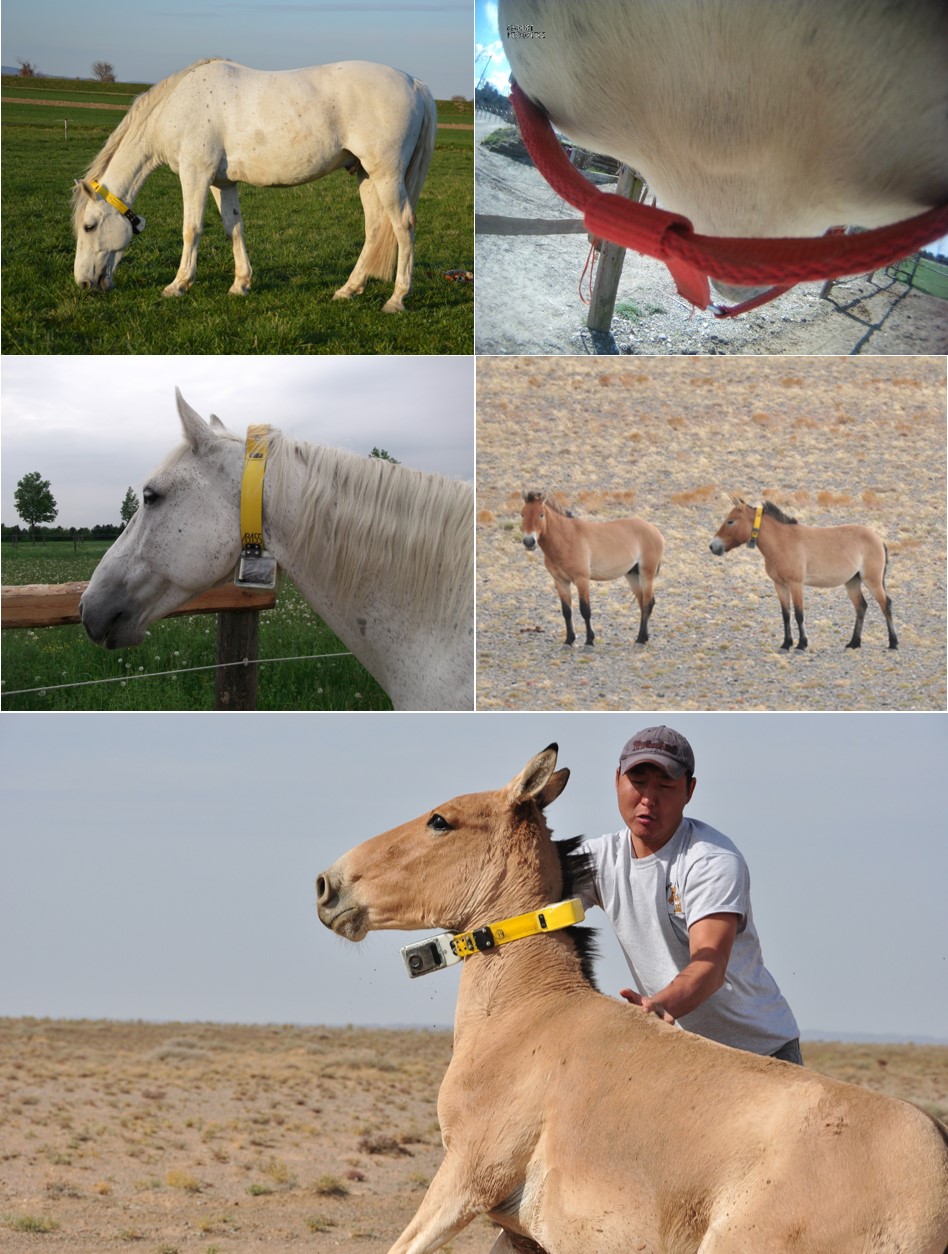
Text.** The camera unit adds substantial to the dimensions and weight of a regular satellite collar requiring thorough testing of camera collar fit and functionality. The original camera collar design had a forward-facing camera, which resulted in images with the lower jaw filling most of the picture. The first alternative with a side-facing camera had a casing which was too wide, restricting the horses’ ability to bend its head and bumping against the mandibles. The next camera collar design had a narrow but long shape which fitted well between the mandibles (Figure A in S1 File).

***S1 Figure.*** *Top left: Original design with forward-facing camera; top right: Resulting image largely obstructed by the lower jaw; middle left: The alternative casing with a sideways facing camera was too wide, resulting in a poor fit (Photos: A. Haymerle; Copyright granted under a CC BY license); middle right: Przewalski’s horse with final camera collar casing in the Mongolian Gobi (Photo: P. Kaczensky); Bottom: The collared khulan with the final camera collar design recovering from anesthesia on 16 October 2015 (Photo: P. Kaczensky; The individual visible in the image has given written informed consent (as outlined in the PLOS consent form) to publish this image).*
