# Supplementary material for: Through the eye of a Gobi khulan – Application of camera collars for ecological research of far-ranging species in remote and highly variable ecosystems
Source: PLoS One. 2019 Jun 4;14(6):e0217772. doi: 10.1371/journal.pone.0217772 (PMC6548383; doi:10.1371/journal.pone.0217772)
Supplement: S2 File — (DOCX) [file pone.0217772.s004.docx]

## S2 File. Coding instructions and illustrations.

***S2 Table****: Coding instructions.*

| **Category** | **Coding** |
| --- | --- |
| Black / Blurry / White | Black = image totally black or at least so black you don’t see anything [*but mark: i) moon (light in the sky) or 2) other (artificial lights]*]  White = image predominantly white [*likely either sky, into the sun, or faulty exposure*]  Blurry = image so burry, that no khulan or habitat information can be extracted |
| Collared khulan seen | NA / yes = parts of her body seen [*more than just some hair or part of a leg – basically enough to have some information about the collared khulan*] |
| Collared khulan shadow seen | NA / yes = shadow [*more than just shadow of a leg – basically enough to have some information value*] |
| Additional shadow animals | NA / yes = shadows suggest another khulan next to the collared khulan [*use the shadow also for “other Khulan in close proximity” if the two shadows are suggesting they are almost touching*] |
| How many other khulan can be counted | Number:   - count only clearly visible khulan [*if there are more in the back fill those in the category “More in the background”*] - add also other khulan which are only seen as shadows [*List only those, which are not otherwise seen in flesh; e.g. if her shadow and the shadow of her foal is seen this value is: 1*] |
| How many foals can be counted | Number: count only those clearly identifiable as foals *[It is O.K. if there are more – this will be a minimum estimate]* |
| How many khulan in close proximity (within one khulan length) | Number:  defined by size of the khulan in the image - any khulan imagined standing (for those laying) that would cover near to ≥one quarter of the total image |
| More in the background (not really countable) | Number: Estimated number (~10) or if this is not possible*:*   - few - many - unable to tell |
| Comments number | Text: only if there is something special |
| Behaviour of collared khulan based on shadow or own body parts visible | - lying down - standing (neck straight) - feeding (neck down) - walking - running - other |
| Comment behaviour collared khulan | Text: only if there is something special |
| ***Bahavior of other khulan for all images with up to 3 khulan clearly visible:*** | |
| Behaviour khulan 1 | - lying down - standing (neck straight) - feeding (neck down) - walking - running - other |
| Behaviour khulan 2 & 3 | Same as Behaviour khulan 1 |
| Comments behaviour for up to 3 khulan clearly seen | Text: only if something special was seen |
| ***Bahaviour of other khulan for all images with >3 khulan clearly visible:*** | |
| Main behaviour (majority of animals is seen doing this) | - lying down - standing (neck straight) - feeding (neck down) - walking - running - other - mixed (*if e.g. 3 khulan feeding and 4 laying)* |
| Comments main behaviour | Text: only if something special |
| Livestock species | Name |
| Livestock number | Number: if countable count, else guess and add "~" (e.g. ~50) |
| Wildlife species | Name |
| Distance wildlife | - close (easily countable) - medium (clearly seen but hard to count)   far (seen in distance) |
| Wildlife number | Number |
| Distance livestock | - close (easily countable) - medium (clearly seen but hard to count) - far (seen in distance) |
| Infrastructure | - Road - Vehicles - Railway embankment - Powerline - Fence - Gers - Buildings - Artificial lights - Other |
| Distance infrastructure | - close (very clear and immediately seen) - medium (intermediate) - far (just able to see) |
| Short description what is seen | Text |
| Habitat type | - Plains - Mountains (hills seen, big rocks) - Dry riverbed (trees, Achnaterum grass, run-off channel) - Waterpoint (water seen, or khulan seen digging, or standing in large number in dry riverbed) - Near water point (sandy hills with Nitraria, achnaterum or trees and many tracks or dung) |
| Weather | - Clear sky - Cloudy (>50%) - Raining (rainfall, wet khulan, rainbow seen) - Snowing - Storm (sandstorm = yellow air, wind storm = tails flying) |
| Evidence of recent rain events | - Raining (rainfall, wet khulan, water on lense, foggy lense & low hanging clouds) - Water on the ground (other than at waterpoint) |
| Snow on the ground | Empty = no snow  <25%  25-50%  >50% |
| Horizon Tilt value | Number in degrees measured with software *ImageJ* (<https://imagej.net/imageJ>) |
| Comment | Anything which was surprising, unusual, or special |

**Coding illustrations**

*
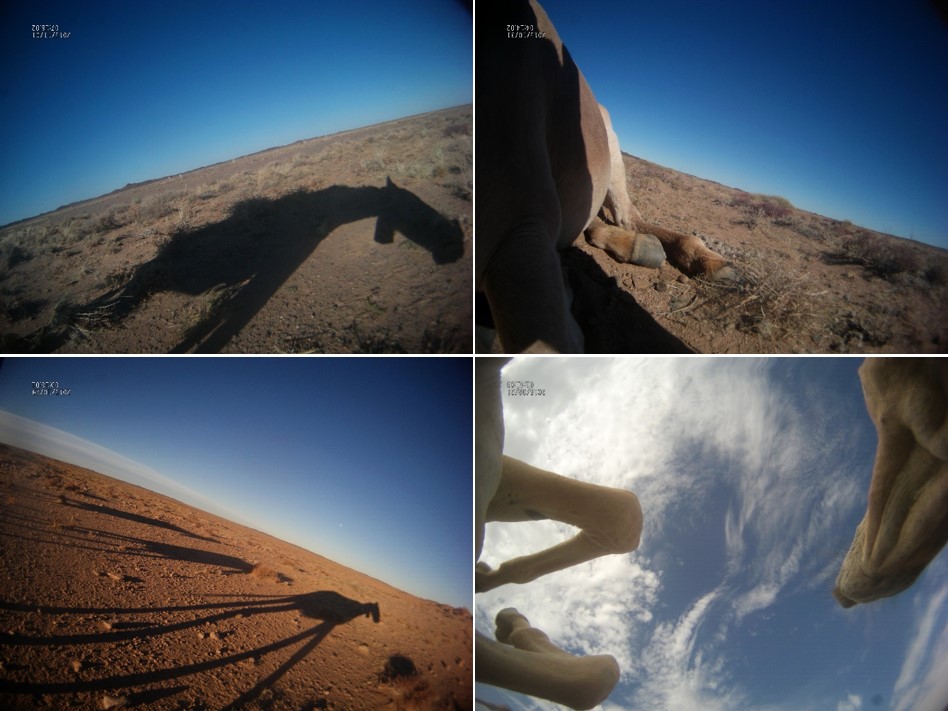
*

***S2 Figure A****. The collared khulan is seen. Top left: Shadow of collared khulan; top right: Body parts of collared khulan; bottom left: Two additional shadow khulan seen (plus 4 additional khulan directly visible in the background); bottom right: Body parts of collared khulan.*

*
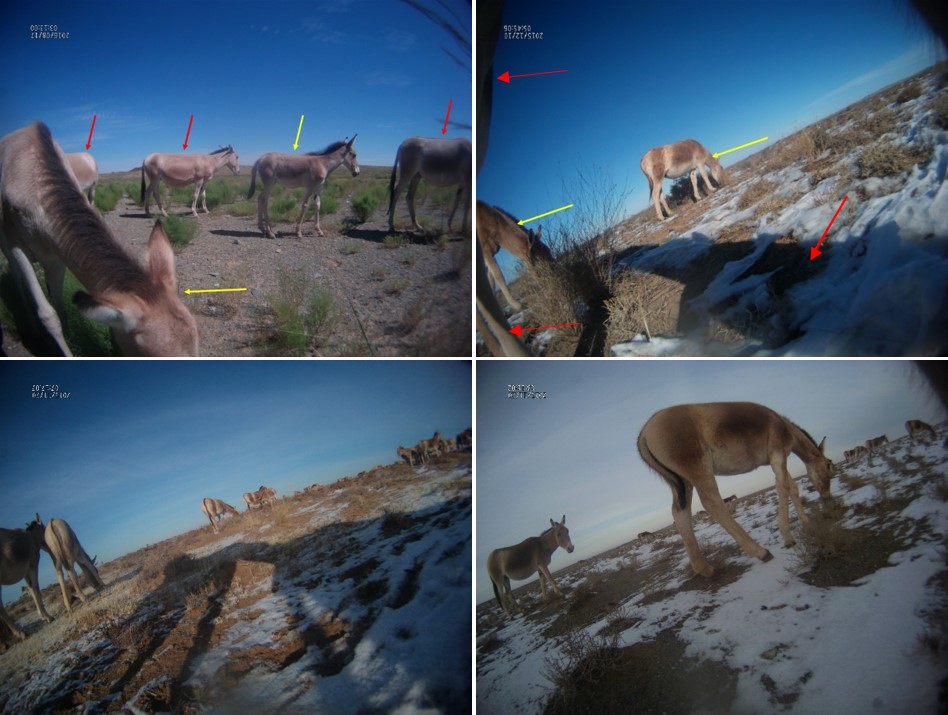
*

***S2 Figure B****. Other khulan seen (including foals and other khulan in close proximity. Top left: Five other khulan seen, of which two are foals (yellow arrows); top right: Two other khulan seen (both foals – yellow arrows), the remaining features and the shadow are from the collared khulan (red arrows); bottom left: 34 khulan counted plus “many” more uncountable in the background (the shadow of the collared khulan is visible in the foreground); bottom right: 19 khulan seen of which one foal is in close proximity, no khulan visible in the background.*

*
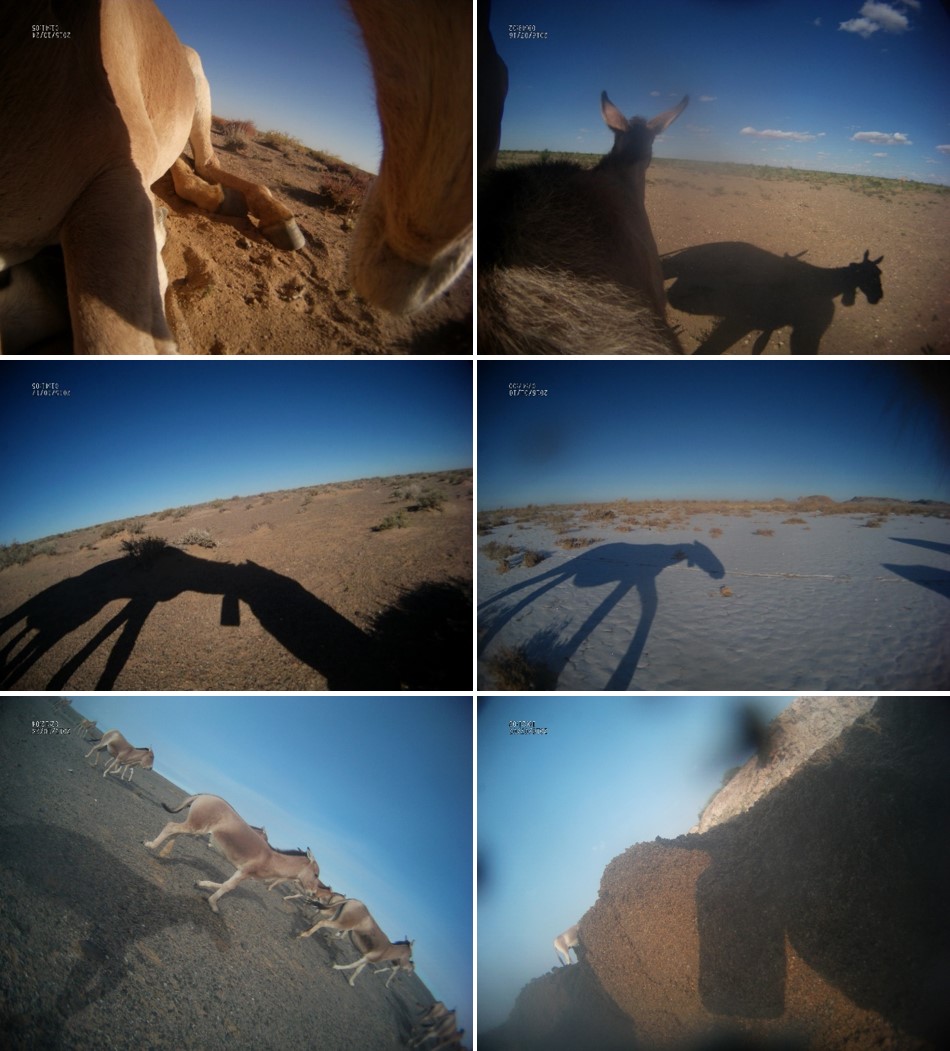
* ***S2 Figure C****. Behaviour of collared khulan. Top left: Lying down; top right: standing (with other khulan – likely foal – close); middle right: feeding on bush; middle right: walking; bottom left: Running; bottom right: Other (most likely drinking as her head is in a hole).*

*
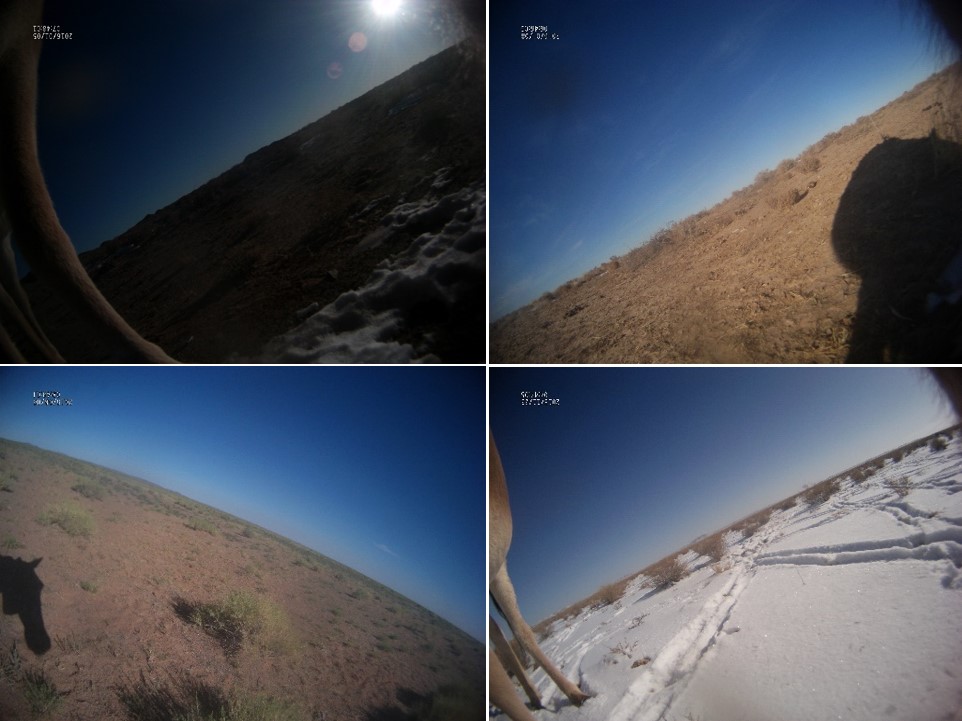
* ***S2 Figure D****. Selection of images that do not show enough of the collared khulan`s body or shadow to allow deducing behaviour.*

*
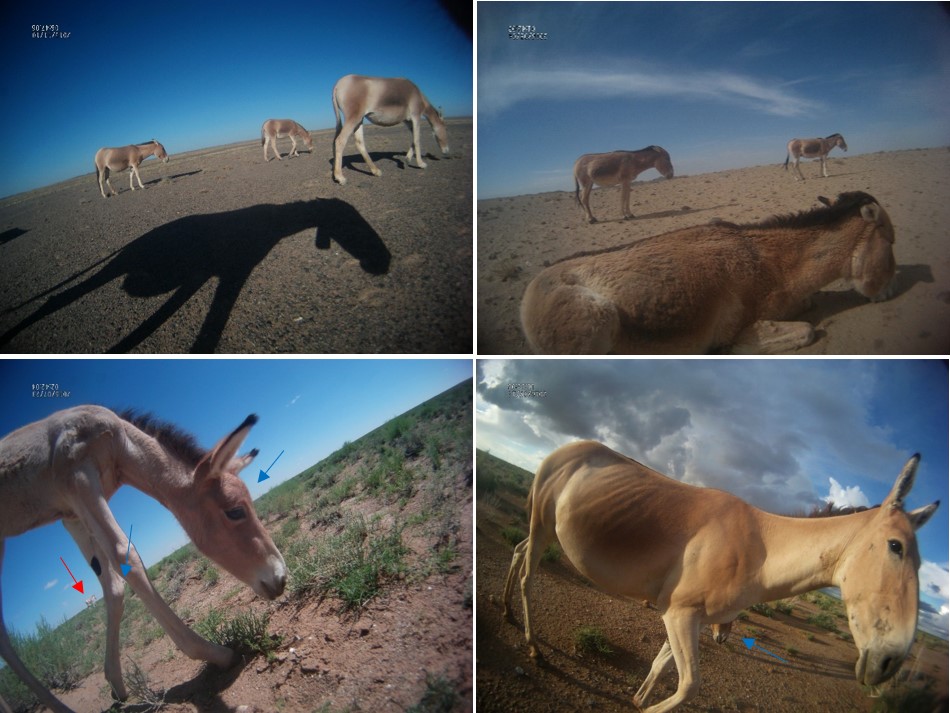
* ***S2 Figure E****. Behaviour of ≤3 khulan. Top left: One standing and two feeding; top right: Two standing, one lying down; bottom left: One standing, two feeding (blue arrows; one khulan is seen in between the foals´ legs with its head down; bottom right: One walking and one feeding (blue arrow).*

*
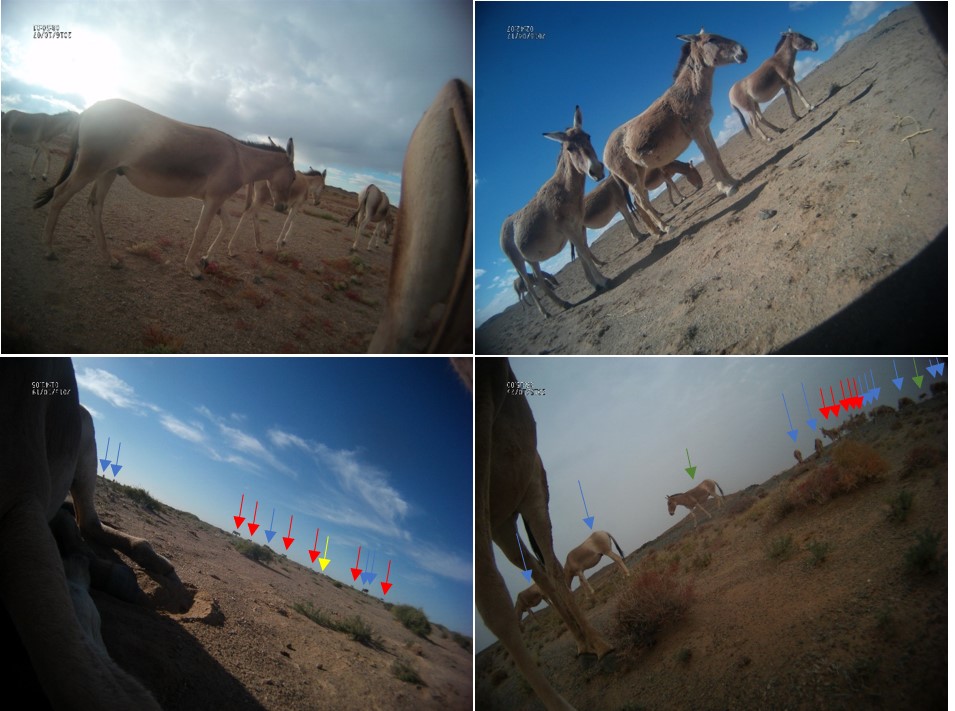
* ***S2 Figure F****. Khulan group behaviour (>3 khulan seen). Top left: Group behavior Walking (all 5 walking); top right: Group behaviour Standing (7 other khulan are seen: 4 standing, 3 feeding); bottom right: Body parts of collared khulan; bottom left: Group behaviour Mixed (12 other khulan are seen in the back: 5 feeding (blue arrow), 6 standing (red arrow), 1 lying down (yellow arrow)); bottom right: Group behavior Feeding (17 other khulan are seen: 10 feeding (blue arrow), 5 standing (red arrows), and 2 walking (green arrows)).*

*
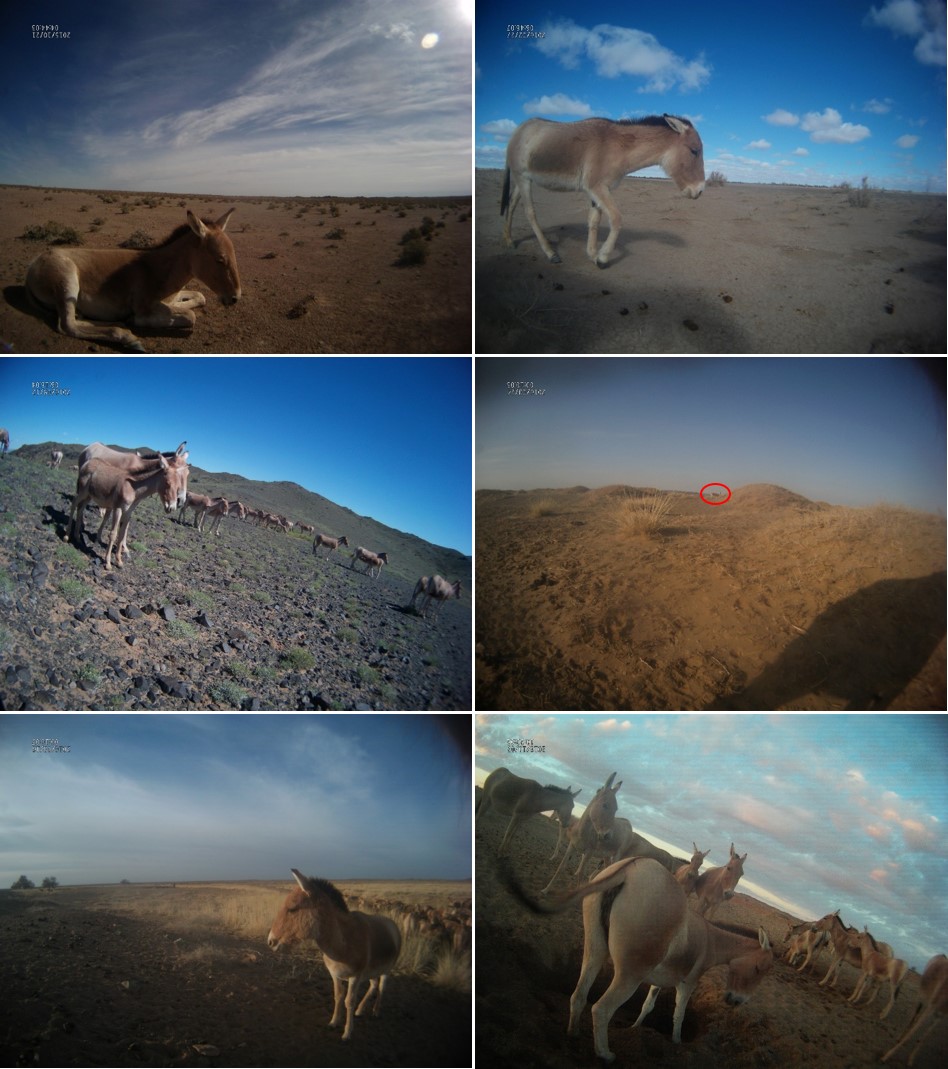
* ***S2 Figure G****. Landscape type. Top left & right: Plains; middle left: hills/mountains; middle right: probably near waterpoint (see large number of tracks and heavily grazed* [*Achnatherum splendens*](https://www.google.no/search?q=Achnatherum+splendens&rlz=1C1GCEU_noKZ821NO821&tbm=isch&tbo=u&source=univ&sa=X&ved=2ahUKEwj6nMmY55DfAhXEwosKHRKxD3wQsAR6BAgDEAE) *grass; within the red circle a gazelle is seen); bottom left: Waterpoint deduced from vegetation, tracks and large number of khulan in dry riverbed); bottom right: Waterpoint showing khulan digging for water.*

*
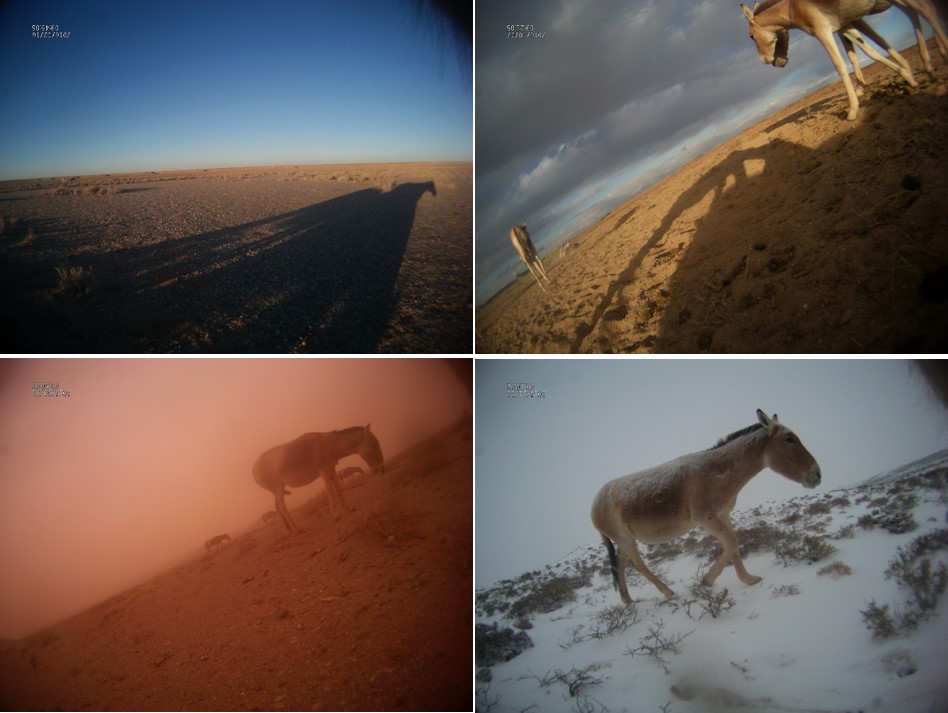
* ***S2 Figure H****. Weather. Top left: Clear sky; top right: Cloudy (>50% cloud cover); bottom left: Sand storm (notice the red color of the image ad how tail is blown between the legs of the khulan in front); bottom right: Snowfall (notice fresh show on the back of the khulan).*

*
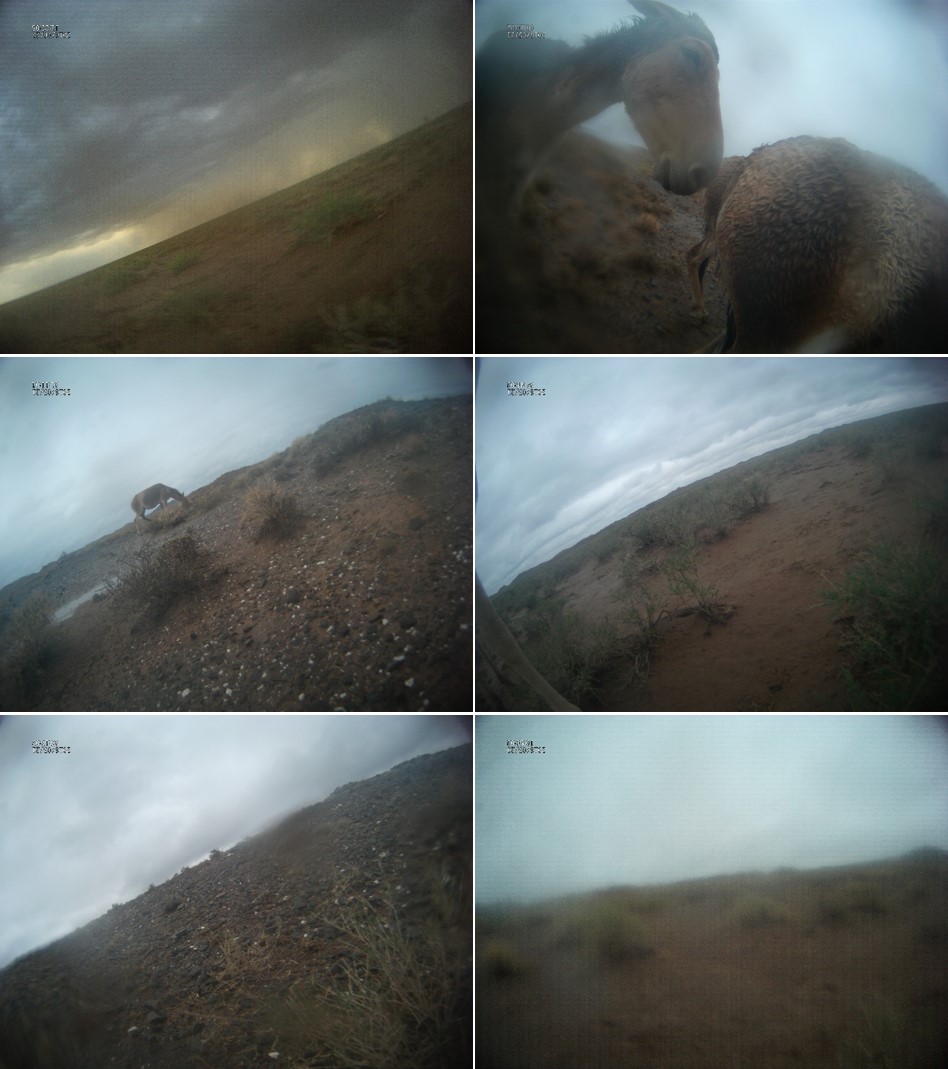
* ***S2 Figure I****. Evidence of rain. Top left: Rainstorm; top right: Rainfall (notice wet fur on khulan); middle right: rainwater on the ground; middle right: wet ground and low hanging clouds; bottom left: water on lens and low hanging clouds; bottom right: fogged up lens and low clouds.*

#

*
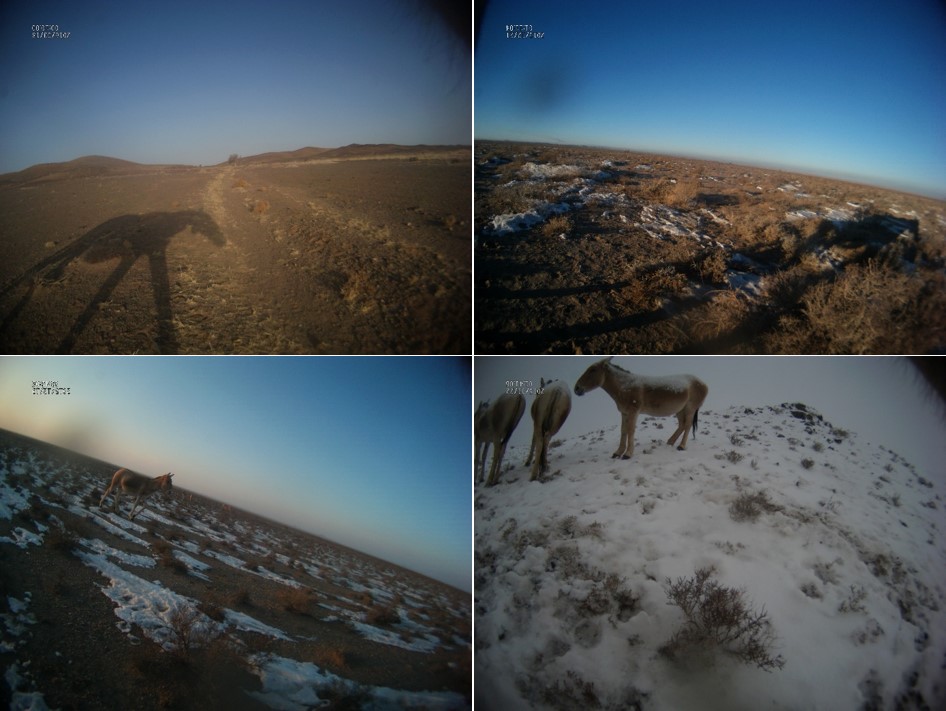
* ***S2 Figure J****. Snow cover. Top left: No snow; top right: Snow cover <25%; bottom left: Snow cover 25-50%; bottom right: Snow cover >50%.*
