# Supplementary material for: Through the eye of a Gobi khulan – Application of camera collars for ecological research of far-ranging species in remote and highly variable ecosystems
Source: PLoS One. 2019 Jun 4;14(6):e0217772. doi: 10.1371/journal.pone.0217772 (PMC6548383; doi:10.1371/journal.pone.0217772)
Supplement: S3 File — (DOCX) [file pone.0217772.s005.docx]

## S3 File. Classification tree.

***S3 Figure****. Supervised classification trees for main behavioural categories based on image tilt and/or collar activity sensor values based on a training set of 277 images of known behaviour.*


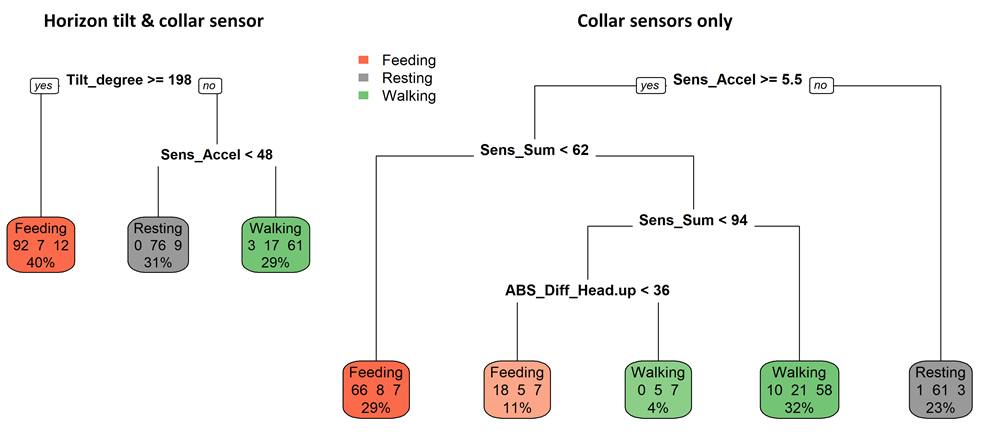


83% 89% 75%

Correctly classified

88% 79% 75%

Correctly classified

*Value codes:* ***Tilt_degree****= tilt of horizon in photograph, in degrees,* ***Sens_Accel****=acceleration sensor value,* ***Sens_Sum****=sum of acceleration and head-up sensor values,* ***ABS_Diff_Head.up****= absolute difference between head-up sensor value in the two intervals proceeding and following the image time stamp. Each box at the leaf ends shows the classification made to the training data set, with a count of categories feeding, resting, and walking reading from left to right. For example, in the leftmost bubble, on the basis of the criterion “horizon tilt degree >=198”, 92 + 7 + 12 = 111 animals, or 40 percent of the total 277, were classified as feeding. Of these, 92 were correctly classified as feeding, 7 were misclassified as resting, and 12 were misclassified as walking.*

***S3 Table A****. Confusion matrix of the two behavioural models.*

|  |  | **Reference** | |  |  |
| --- | --- | --- | --- | --- | --- |
| **Prediction** |  | **Feeding** | **Resting** | **Walking** | **Total** |
| ***Horizon tilt & collar activity sensors*** | | | | |  |
|  | Feeding | 51 | 1 | 3 |  |
|  | Resting | 1 | 42 | 7 |  |
|  | Walking | 12 | 2 | 20 |  |
| Proportion correcly classified | | 0.93 | 0.84 | 0.59 | 0.81 |
| ***Only collar activity sensor*** | | | |  |  |
|  | Feeding | 41 | 1 | 13 |  |
|  | Resting | 6 | 34 | 10 |  |
|  | Walking | 11 | 1 | 22 |  |
| Proportion correcly classified | | 0.75 | 0.68 | 0.65 | 0.70 |

*Training data = 380 observations, test data = 189 observations*

***S3 Table B****. Behavioural categories of the collared khulan based on different methods.*

| **Method** | **Feeding** | **Resting** | **Walking** | **N** | **Data source** |
| --- | --- | --- | --- | --- | --- |
| Hand-coded (day only) | 52.4 | 39.6 | 10.6 | 1,116 | Images of collared khulan |
| Classified - Tilt & sensor model (day only) | 52.1 | 25.9 | 22 | 8,719 | All images |
| Classified - Sensor only model (day only) | 41.9 | 19.7 | 38.3 | 54,879 | 4.8min activity intervals |
| Classified – Collar sensor only model (20:00-7:00) | 42.7 | 20.7 | 36.5 | 54,876 | 4.8min activity intervals |
