# Supplementary material for: Through the eye of a Gobi khulan – Application of camera collars for ecological research of far-ranging species in remote and highly variable ecosystems
Source: PLoS One. 2019 Jun 4;14(6):e0217772. doi: 10.1371/journal.pone.0217772 (PMC6548383; doi:10.1371/journal.pone.0217772)
Supplement: S4 File — (DOCX) [file pone.0217772.s006.docx]

## S4 File. Other khulan seen.

In a total of 32 images, 20 or more khulan were visible and in an additional 10 images “many” more khulan were visible in the background, but could not be reliably counted, making for a total of 42 images (0.5%) with larger khulan aggregations (S6 Fig).


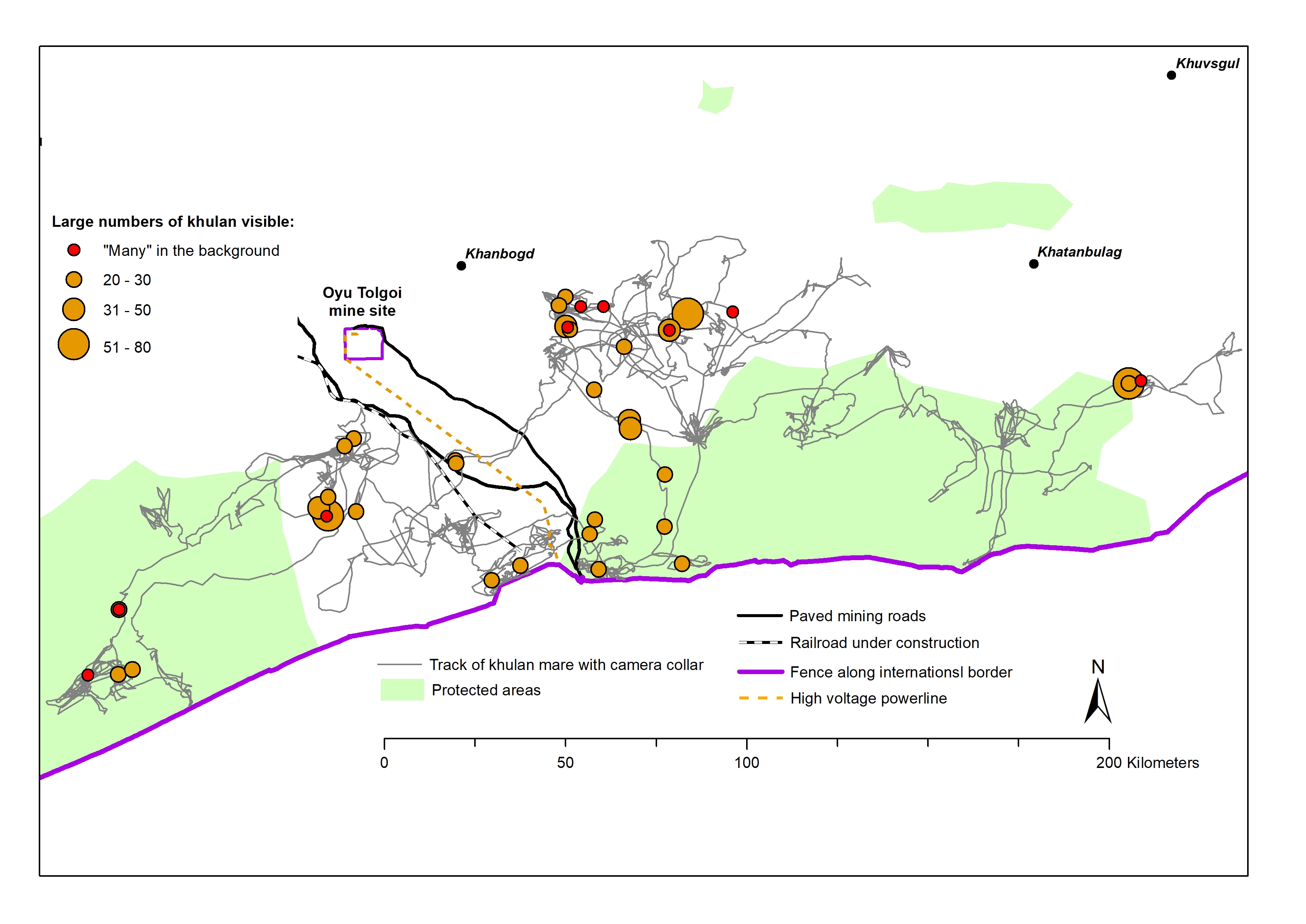


***S4 Figure****. Location of larger khulan aggregations.*

***S4 Table****. Number of images which show other khulan in close proximity.*

| **Year / Month** | **Number of other khulan in close proximity** | | | | **Total** |
| --- | --- | --- | --- | --- | --- |
|  | **1** | **2** | **3** | **4** |  |
| 10 / 2015 | 41* | 4 | 1 |  | 46 |
| 11 / 2015 | 67 | 10 | 2 |  | 78 |
| 12 / 2015 | 53 | 5 | 4 |  | 62 |
| 01 / 2016 | 64 | 13 | 3 |  | 80 |
| 02 / 2016 | 63 | 5 |  |  | 68 |
| 03 / 2016 | 52 | 2 | 2 |  | 56 |
| 04 / 2016 | 16 | 2 | 1 |  | 19 |
| 05 / 2016 | 26 | 4 | 1 |  | 31 |
| 06 / 2016 | 5 | 1 |  |  | 6 |
| 07 / 2016 | 84 | 5 |  |  | 89 |
| 08 / 2016 | 86 | 9 | 2 | 2 | 99 |
| 09 / 2016 | 91 | 3 |  |  | 94 |
| 10 / 2016 | 30* | 6 |  |  | 36 |
| **Total** | **678** | **69** | **16** | **2** | **765** |

**Camera collared was only operational for half the months, due to deployment on*

*16.10.2015 and drop on 16.10.2016.*
