# Supplementary material for: Through the eye of a Gobi khulan – Application of camera collars for ecological research of far-ranging species in remote and highly variable ecosystems
Source: PLoS One. 2019 Jun 4;14(6):e0217772. doi: 10.1371/journal.pone.0217772 (PMC6548383; doi:10.1371/journal.pone.0217772)
Supplement: S5 File — (DOCX) [file pone.0217772.s007.docx]

## S5 File. Weather conditions.

***S5 Table****. Weather conditions based on 7,854 camera collar images.*

| **Year / Month** | **% Images** | | | | |
| --- | --- | --- | --- | --- | --- |
|  | **Sunny** | **Cloudy** | **Raining** | **Snowing** | **Sandstorm** |
| 2015/10 | 79.9 | 20.1 |  |  |  |
| 2015/11 | 63.0 | 34.8 |  | 2.2 |  |
| 2015/12 | 80.3 | 19.7 |  |  |  |
| 2016/01 | 82.2 | 17.8 |  |  |  |
| 2016/02 | 82.2 | 17.8 |  |  |  |
| 2016/03 | 61.3 | 36.2 |  |  | 2.5 |
| 2016/04 | 65.4 | 32.6 |  |  | 2.0 |
| 2016/05 | 51.3 | 38.5 | 3.1 |  | 7.2 |
| 2016/06 | 50.7 | 46.1 | 3.2 |  |  |
| 2016/07 | 60.5 | 38.2 | 1.4 |  |  |
| 2016/08 | 68.2 | 31.1 | 0.7 |  |  |
| 2016/09 | 61.3 | 38.0 | 0.6 |  |  |
| 2016/10 | 72.6 | 27.4 |  |  |  |


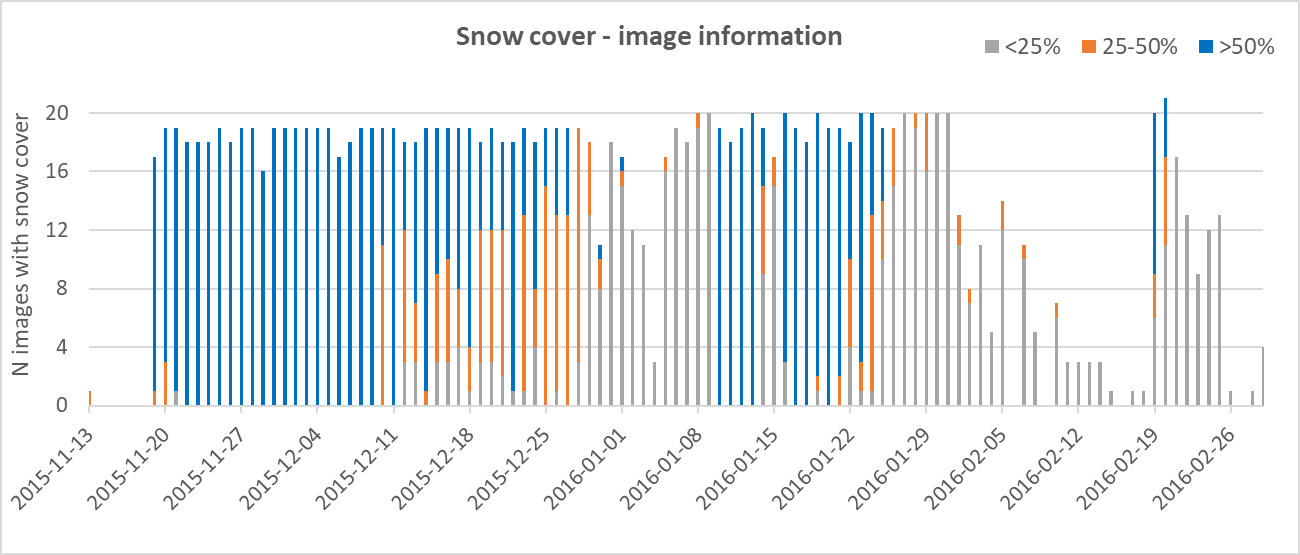


***S5 Figure****. Quantitative snow cover information from camera collar images.*

## 
