# Supplementary material for: Through the eye of a Gobi khulan – Application of camera collars for ecological research of far-ranging species in remote and highly variable ecosystems
Source: PLoS One. 2019 Jun 4;14(6):e0217772. doi: 10.1371/journal.pone.0217772 (PMC6548383; doi:10.1371/journal.pone.0217772)
Supplement: S1 Fig — (DOCX) [file pone.0217772.s008.docx]

## S1 Fig. Image tilt and activity sensor values of main behavioural categories.

***S1 Fig****. Mean values for image tilt and activity sensor values for images (N=1,630) showing the behaviour of the collared khulan.*
