# Supplementary material for: Through the eye of a Gobi khulan – Application of camera collars for ecological research of far-ranging species in remote and highly variable ecosystems
Source: PLoS One. 2019 Jun 4;14(6):e0217772. doi: 10.1371/journal.pone.0217772 (PMC6548383; doi:10.1371/journal.pone.0217772)
Supplement: S2 Fig — (DOCX) [file pone.0217772.s009.docx]

## S2 Fig. Climatic conditions October 2015 - October 2016.

Gap in

snow data


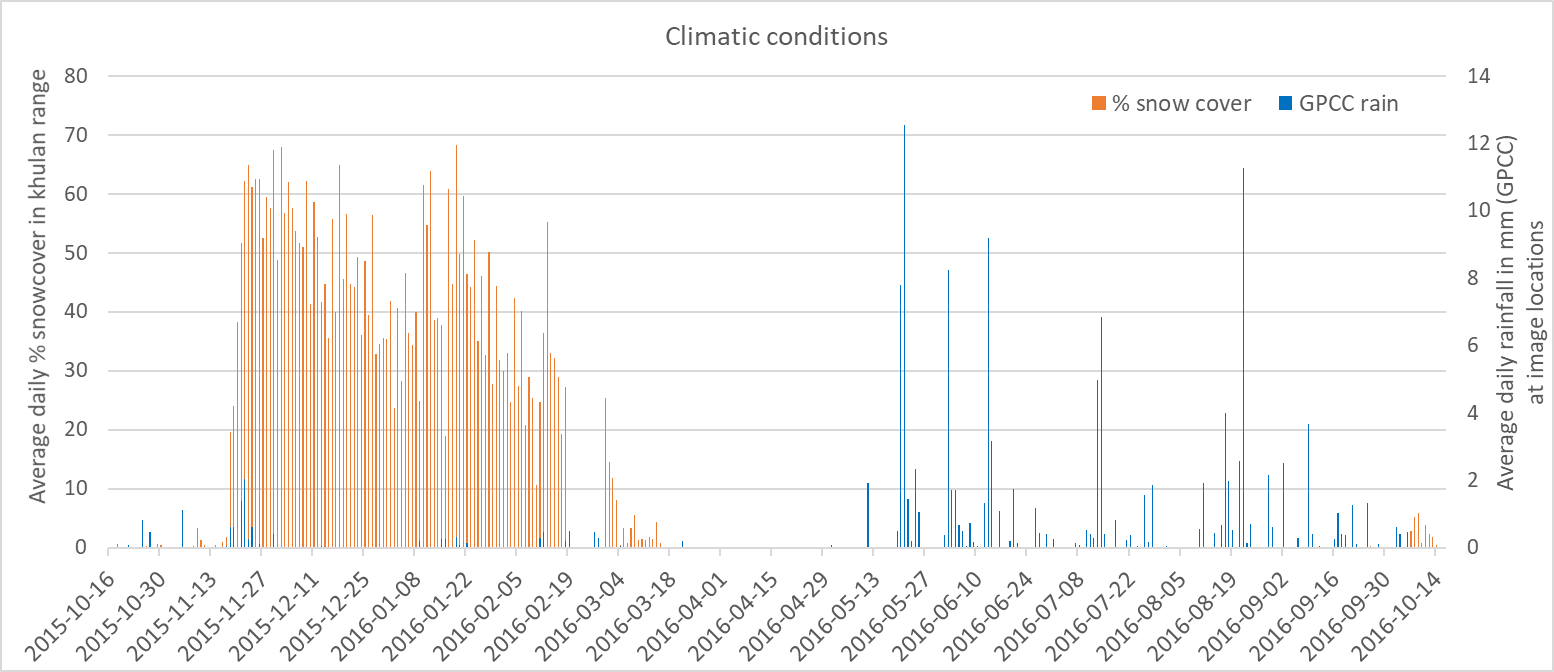


***S2 Figure****. Daily snow cover based on the MODIS/Terra Snow Cover daily product (note gap in data from 19-28 Feb 2016) and precipitation (mm) based on the GPCC First Guess daily product for the monitoring period 16 Oct 2015 – 16 Oct 2016.*
