# Supplementary material for: Through the eye of a Gobi khulan – Application of camera collars for ecological research of far-ranging species in remote and highly variable ecosystems
Source: PLoS One. 2019 Jun 4;14(6):e0217772. doi: 10.1371/journal.pone.0217772 (PMC6548383; doi:10.1371/journal.pone.0217772)
Supplement: S3 Fig — (DOCX) [file pone.0217772.s010.docx]

## *
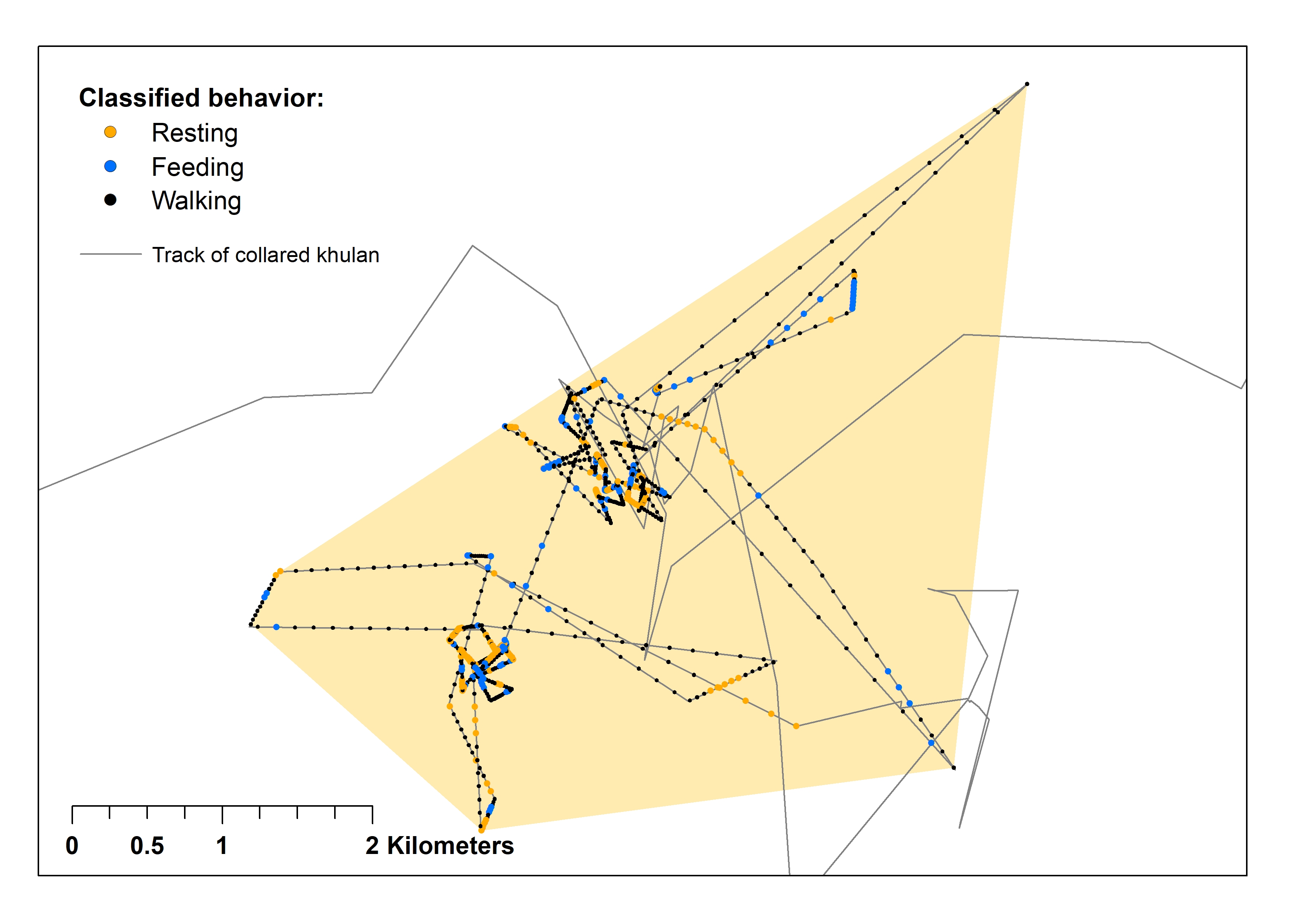
*S3 Fig. Movements and behavior around foaling.

##

***S3 Figure****. Spatially confined movements during the first 4 days post-partum. Activity readings show a high proportion of walking which is likely the result of a high activity other behavior related to taking care and interacting with a newborn.*
